# R-spondin3 is a myokine that differentiates myoblasts to type I fibres

**DOI:** 10.1038/s41598-022-16640-2

**Published:** 2022-07-29

**Authors:** Yoshitaka Mita, Haonan Zhu, Yasuro Furuichi, Hiroki Hamaguchi, Yasuko Manabe, Nobuharu L. Fujii

**Affiliations:** Department of Health Promotion Sciences, Graduate School of Human Health Sciences, Tokyo Metropolitan University, 1-1 Minami-Osawa, Hachioji, Tokyo, 192-0397 Japan

**Keywords:** Extracellular signalling molecules, Muscle stem cells

## Abstract

Muscle fibres are broadly categorised into types I and II; the fibre-type ratio determines the contractile and metabolic properties of skeletal muscle tissue. The maintenance of type I fibres is essential for the prevention of obesity and the treatment of muscle atrophy caused by type 2 diabetes or unloading. Some reports suggest that myokines are related to muscle fibre type determination. We thus explored whether a myokine determines whether satellite cells differentiate to type I fibres. By examining the fibre types separately, we identified R-spondin 3 (Rspo3) as a myokine of interest, a secreted protein known as an activator of Wnt signalling pathways. To examine whether Rspo3 induces type I fibres, primary myoblasts prepared from mouse soleus muscles were exposed to a differentiation medium containing the mouse recombinant Rspo3 protein. Expression of myosin heavy chain (MyHC) I, a marker of type I fibre, significantly increased in the differentiated myotubes compared with a control. The Wnt/β-catenin pathway was shown to be the dominant signalling pathway which induces Rspo3-induced MyHC I expression. These results revealed Rspo3 as a myokine that determines whether satellite cells differentiate to type I fibres.

## Introduction

Skeletal muscles play critical roles not only in physical activity and metabolic homeostasis, but also in regulating the functions of distal organs by secreting bioactive molecules called myokines. Much attention has been directed to myokines as key players in the derivation of health benefits from exercise^1^. Some myokines act locally within skeletal muscle tissue and regulate both muscle volume and quality^2^: myostatin/growth differentiation factor 8 (GDF-8) has been long known as a negative regulator of muscle mass^3,4^; interleukin-6 (IL-6) and macrophage migration inhibitory factor (MIF), exercise-regulated myokines, are recognised as working to activate fatty acid metabolism or glucose uptake in skeletal muscle^5,6^. In contrast, several myokines such as insulin-like growth factor 1 (IGF-1), leukemia inhibitory factor (LIF) and glyceraldehyde-3-phosphate dehydrogenase (GAPDH) are known to act in a paracrine manner, acting on surrounding muscle-specific stem cells known as satellite cells, and regulate muscle regeneration^7–10^. Thus, myokines are required for the maintenance of muscle mass and quality, and act in an autocrine and/or paracrine manner.

Muscle fibre is broadly categorised into two types, slow- and fast-twitch fibres. Slow-twitch fibres (also called type I fibres) express myosin heavy chain (MyHC) I and are characterised by fatigue resistance and a high oxidative capacity. Fast-twitch fibres (also called type II fibres) express MyHC II and are characterised by a high glycolytic property, power, and speed, but low endurance. The fibre-type ratio determines the contractile and metabolic properties of skeletal muscle tissue. Although it has long been believed that fibre-type composition is genetically determined, some previous studies argued that the variance in the proportion of type I muscle fibres was influenced not only by inherited factors but also by environmental factors^11^. In fact, some studies have shown that muscle fibre composition is quite plastic and is affected by various factors such as endurance exercise^12,13^ or metabolic diseases such as obesity and diabetes^14^. For example, obese subjects have fewer type I fibres and more type IIb fibres than lean subjects^15^; this has also been shown to be involved in the progression of metabolic disorders^16,17^. Therefore, elucidating the factors and mechanisms that maintain and generate type I fibres promises to be beneficial for the maintenance of health.

Recent studies suggest that properties of muscle fibre are regulated by myokines in an auto/paracrine manner^18,19^. For example, muscle-derived brain derived neurotrophic factor (BDNF) was reported as a regulator for type II fibre specification^19^. Since the features of type I fibres differ so much from those of type II fibres, it can be easily speculated that regulating factors exist which are distinct between the two fibre types. Therefore, in order to identify a type I determination factor, investigations are necessary which allow a specific separation of type I and type II fibres. For this purpose, we used transgenic mice expressing cyan fluorescent protein (CFP) gene under a MyHC I promotor^20^. Only type I fibres can yield a blue fluorescence in the mouse muscles, not type II fibres, allowing us to investigate them separately. Using this method, we found that R-spondin 3 (Rspo3) is specifically expressed in type I fibres. This result motivated us to focus on Rspo3. Finally, we discovered that Rspo3 is a novel myokine that is specifically expressed in type I fibres, affecting satellite cells in a paracrine manner and guiding them to differentiate to type I fibres via Wnt/β-catenin signalling.

## Results

### Rspo3 is specifically expressed in type I fibres

To collect type I and type II muscle fibres separately from soleus (SOL) muscle tissues (consisting of type I and type II fibres equally^21^), we utilised a transgenic mouse (Myh7-CFP mouse) in which only type I fibres are labelled with CFP fluorescence (Supplementary Fig. 1)^20^. Muscle tissues from transgenic mice were treated with collagenase to isolate each fibre; they were then checked for CFP fluorescence with a fluorescent stereomicroscope. CFP positive and negative muscle fibres were pooled and analysed for subsequent experiments.

**Figure 1:**
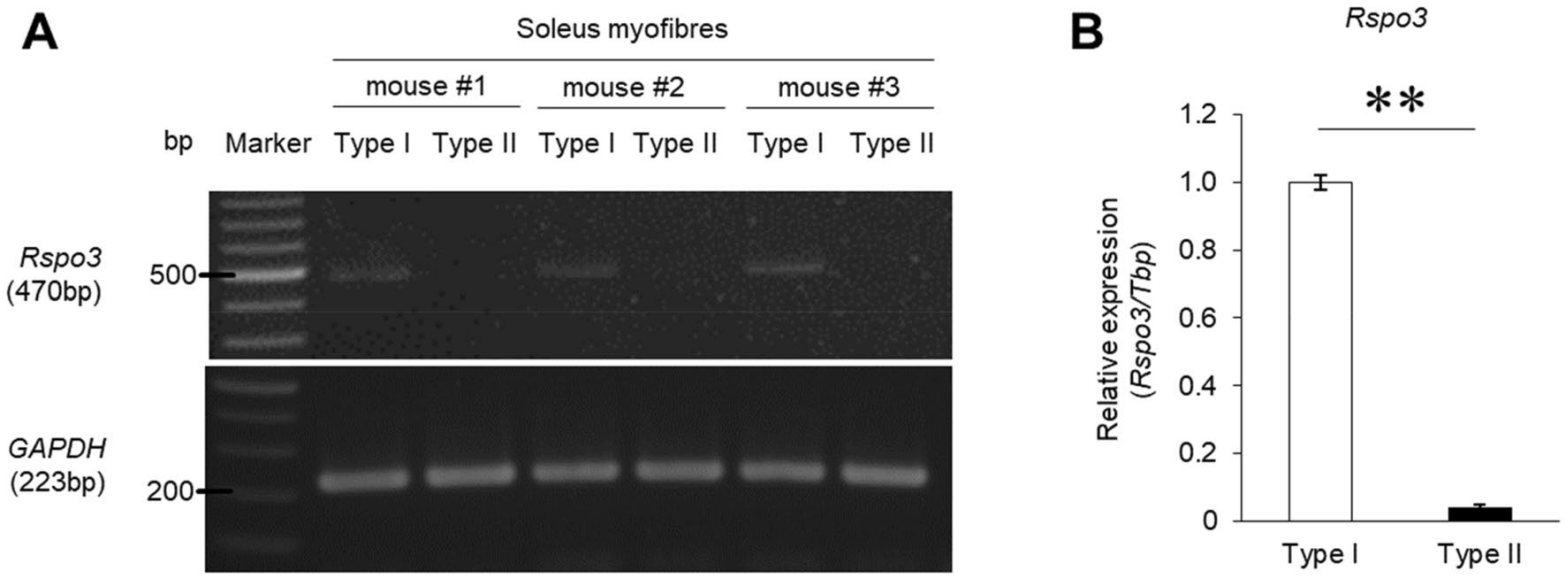
*Rspo3* is specifically expressed in type I fibres. (**A**) The mRNA expression of the *Rspo3* gene was analysed in type I and II fibres (n = 3) using conventional RT-PCR. (**B**) The mRNA expression level of *Rspo3* in type I and II fibres was quantified by quantitative RT-PCR analysis. Values are presented as mean ± SEM (n = 3 mice). **P < 0.01 by Student’s t-test. Uncropped gels can be found in Supplementary Fig. 8.

We took particular note of Rspo3, a secreted protein. Previous studies reported that the Rspo family acting through Wnt signalling pathways are key positive regulators of skeletal myogenesis^22^, and regulate the formation of myotubes^23^. Conventional RT-PCR detected expression of *Rspo3* mRNA only in type I fibres, not in type II fibres (Fig. 1A); other members of the Rspo family are not expressed in the same way in skeletal muscle cells (see Supplementary Fig. 2). Real time quantitative RT-PCR revealed that the *Rspo3* expression level was 25 times higher in type I fibres compared with type II fibres (Fig. 1B). Although *Rspo3* was reported to be expressed in myotubes^22^ and skeletal muscle tissue^24^, no studies have reported *Rspo3* expression in skeletal muscle fibres. In fact, *Rspo3* expression levels are very low in skeletal muscle tissue mainly containing type II fibres (tibialis anterior (TA) and extensor digitorum longus (EDL) muscle), particularly compared with those containing a significant proportion of type I fibres (SOL) (Supplementary Fig. 3). This is probably because other cells in skeletal muscle tissue which are not type I fibres exhibit a much lower level of expression.

### Rspo3 induces expression of MyHC I during myogenic differentiation of primary myotubes

**Figure 2:**
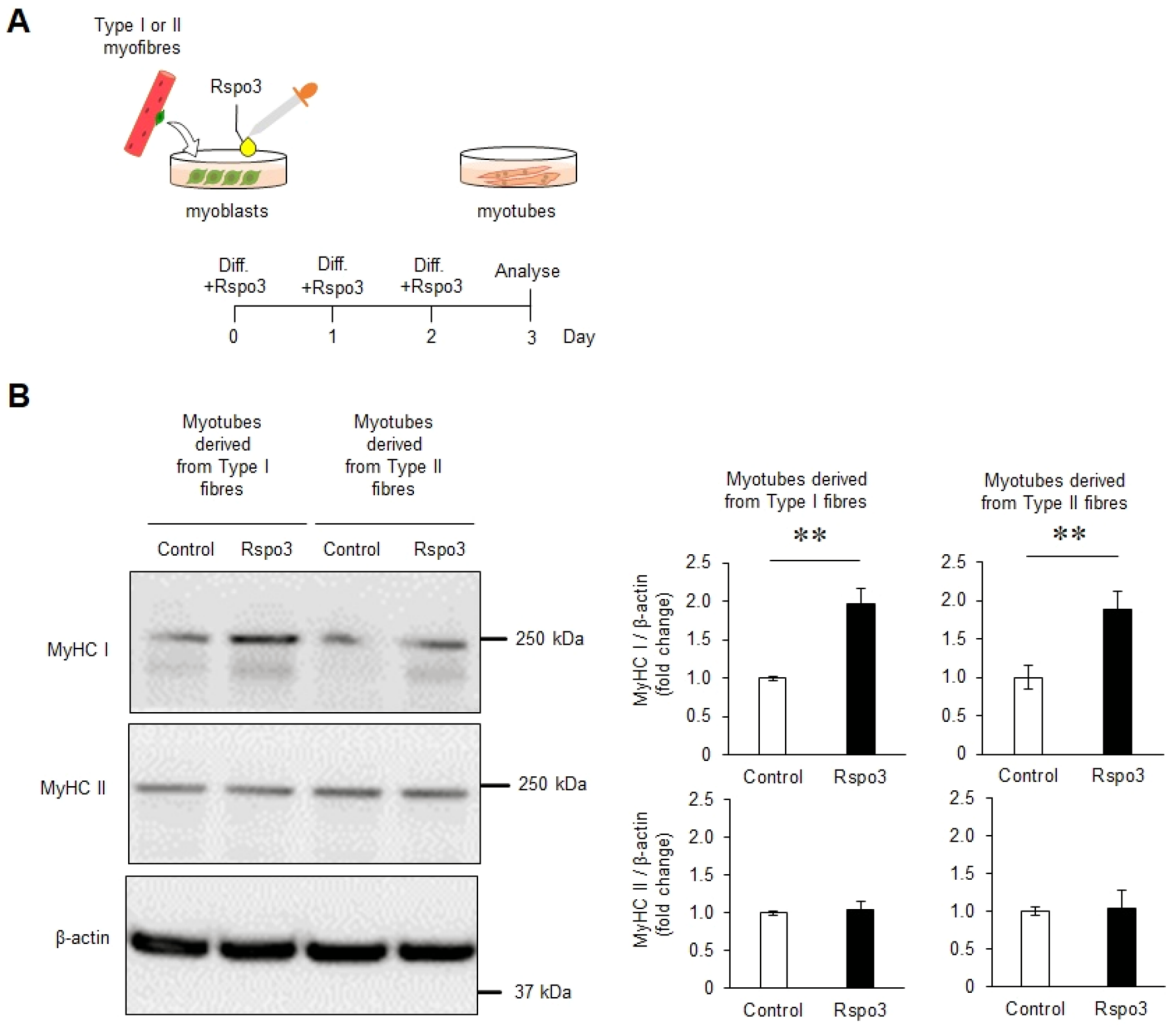
Rspo3 treatment increases MyHC I expression in myotubes. (**A**) Experimental scheme. Mouse primary myoblasts derived from satellite cells of type I or type II fibres were cultured for 3 d in a differentiation media containing BSA (200 ng/mL; Control) or mouse Rspo3 recombinant protein (200 ng/mL; 6.45 nM). (**B**) Protein levels of MyHC I and MyHC II in the differentiated myotubes were measured by Western blotting. The expression of these proteins was normalised to that of β-actin. Values are presented as mean ± SEM (n = 6 mice for analysing MyHC I, n = 4–5 mice for analysing MyHC II). **P < 0.01 by Student’s t-test. Uncropped blots can be found in Supplementary Fig. 9.

To examine whether Rspo3 decides the fate of satellite cells i.e. whether they differentiate to type I fibres, primary myoblasts were treated with mouse Rspo3 recombinant protein (mRspo3). This experiment was done using myoblasts which were derived from both type I fibres (CFP + muscle fibres) and type II fibres (CFP-). Myoblasts were treated with mRspo3 from the start of differentiation for 72 h, and the protein expressions of MyHC isoforms were quantified by western blotting (Fig. 2A). The expression of MyHC I was significantly increased in mRspo3 treated myotubes derived from both types of myoblasts compared with control myoblasts, which were not treated with mRspo3, whereas expression of MyHC II was not affected by the mRspo3 treatment (Fig. 2B). Additionally, differentiated myotubes from myoblasts derived from the type I and type II fibres were treated with mRspo3 for 72 h, starting 3 days after the start of differentiation. However, no change in MyHC I and MyHC II expression was seen in both types of differentiated myotube (Supplementary Fig. 4). These data suggest that Rspo3 acts on myotubes in the early stage of differentiation to generate type I fibres.

### Rspo3 enhances Wnt/β-catenin signalling pathway during muscle differentiation

R-spondin family members have been reported to enhance Wnt signalling pathways, both canonical (Wnt/β-catenin) and non-canonical (Wnt/Planar Cell Polarity (PCP) and Wnt/calcium)^25^. Although Wnt signalling pathways are crucial regulators of myogenesis^26^, the required pathway is distinct depending on the phase of myogenesis. For example, the Wnt/β-catenin signalling pathway promotes myogenic differentiation in muscle progenitor cells^27^ while the Wnt/PCP signalling pathway controls the homeostatic level of satellite cells^28^. To determine which Wnt signalling pathways are regulated by Rspo3 during myogenic differentiation, we treated myoblasts with mRspo3 from the start of differentiation for 72 h (see Fig. 2A) and investigated which Wnt signalling pathway is activated.

**Figure 3:**
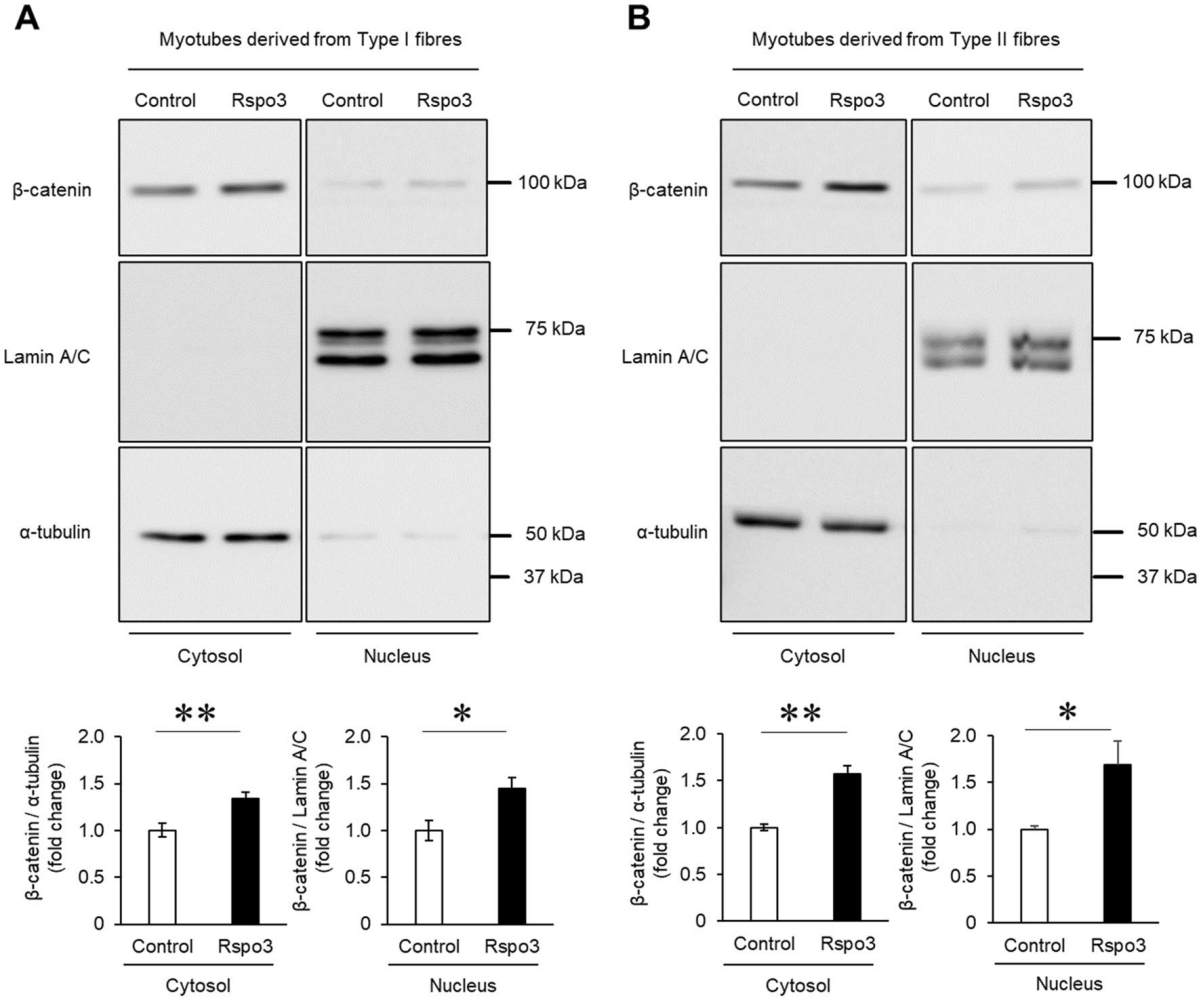
Rspo3 activates the Wnt/β-catenin signalling pathway during myogenic differentiation. (**A**, **B**) Mouse primary myoblasts derived from satellite cells of type I and type II fibres were differentiated for 3 d in the absence (BSA; 200 ng/mL) or presence of the mouse Rspo3 recombinant protein (200 ng/mL; 6.45 nM). Cytoplasmic and nucleic fractions were extracted from myotube lysate. Protein levels of β-catenin, lamin A/C (nuclear marker), and α-tubulin (cytosol marker) in myotubes were analysed using Western blotting. Values are presented as mean ± SEM (n = 10–12 mice for analysing cells derived from type I fibres, n = 8 mice for analysing cells derived from type II fibres). *P < 0.05; **P < 0.01 by Student’s t-test. Uncropped blots can be found in Supplementary Fig. 10.

**Figure 4:**
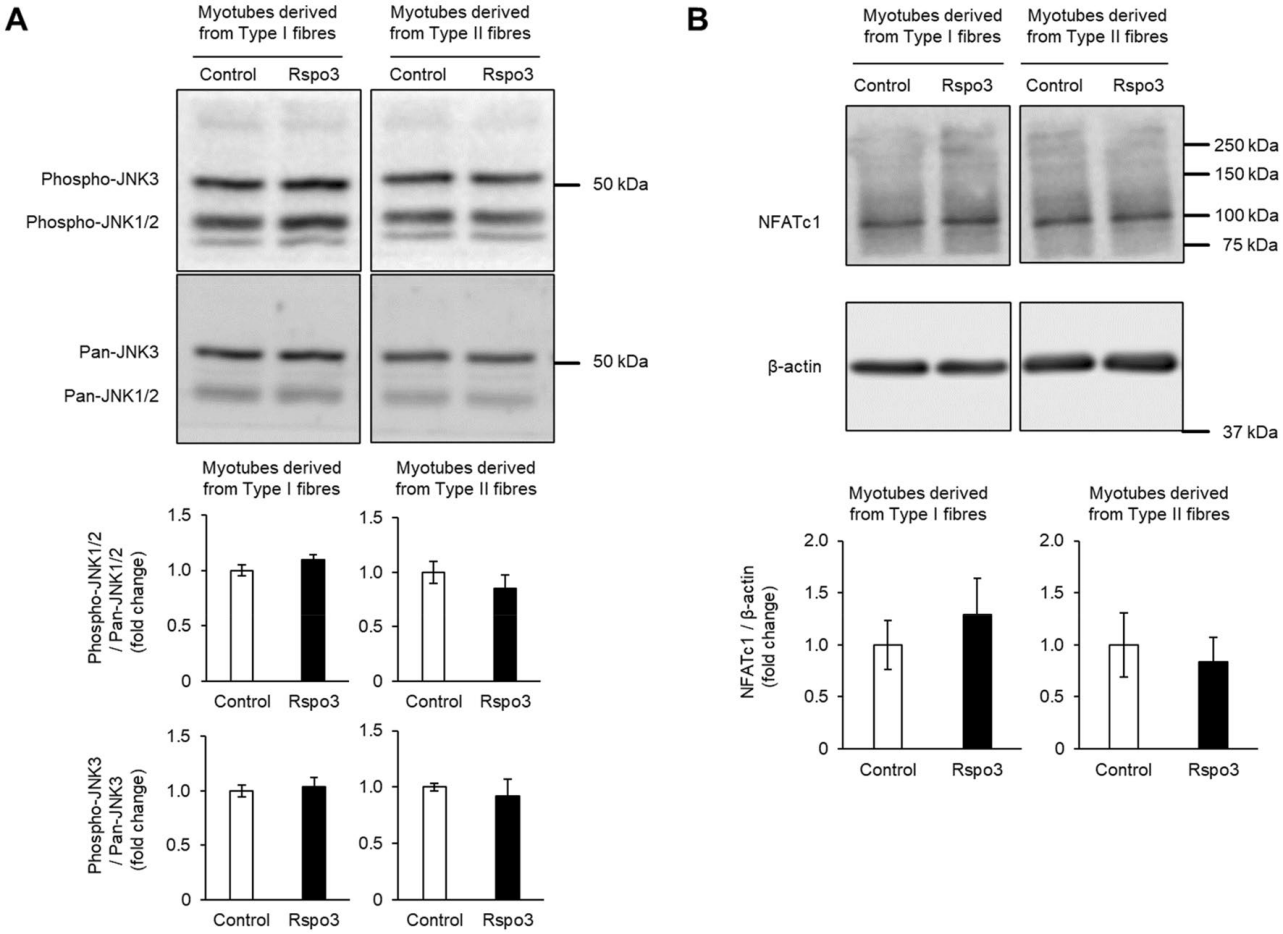
Rspo3 does not activate non-canonical Wnt signalling pathways during myogenic differentiation. (**A**) Mouse primary myoblasts derived from satellite cells of type I and type II fibres were differentiated for 3 d in the absence (BSA; 200 ng/mL) or presence of the mouse Rspo3 recombinant protein (200 ng/mL; 6.45 nM). Protein levels of phospho-JNK1/2/3 and pan-JNK1/2/3 in myotubes were analysed using Western blotting. Values are presented as mean ± SEM (n = 6 mice). (**B**) Mouse primary myoblasts derived from satellite cells of type I and type II fibres were cultured for 3 d in the differentiation media as described in (**A**). Protein levels of NFATc1 in myotubes were analysed using Western blotting. Values are presented as mean ± SEM (n = 12 mice for analysing cells derived from type I fibres, n = 10 mice for analysing cells derived from type II fibres). Uncropped blots can be found in Supplementary Fig. 11.

Once the Wnt/β-catenin signalling pathway is activated, β-catenin accumulates in the cytoplasmic side through a degradation blockade, where conformational changes occur in the destruction complexes which degrade β-catenin^29^. Stabilised β-catenin in the cytosol then translocates into the nucleus and binds members of transcription factors to induce Wnt/β-catenin target genes. The amount of β-catenin in the nuclei of myotubes was significantly increased by mRpso3 treatment regardless of the type of fibre from which the satellite cells derive (Fig. 3A,B), and increased in a dose dependent manner (Supplementary Fig. 5). We observed bare bands of α-tubulin in the nuclear fraction in both myoblasts derived from type I and type II fibres. This might be contamination of the cytosol fraction; however, despite this, we judged that it did not affect our result because the amount of contamination was low. This suggests that Rspo3 treatment results in the increase of β-catenin translocation into the nucleus. Next, we determined whether Rspo3 enhances non-canonical Wnt signalling pathways (Wnt/PCP and Wnt/calcium signalling pathway). Wnt/PCP components can result in the activation of small GTPases Rac and Rho, leading to induction of the phosphorylation of c-Jun NH2-terminal kinase (JNK) to activate Wnt/PCP target genes^30^. However, phosphorylation of JNK 1/2/3 did not change with mRspo3 treatment (Fig. 4A). We also note that activation of the Wnt/calcium signalling pathway would increase intracellular Ca^2+^ levels; calcineurin is then activated, followed by the activation of its target, the nuclear factor of activated T-cells (NFAT), where NFAT is dephosphorylated and its nuclear localization signal is unmasked^31^. Because NFATc1 is involved in slow skeletal muscle gene expression^32^, we focused on the abundance of NFATc1 in differentiated myotubes treated with mRspo3. However, multiple bands indicating the phosphorylation of NFATc1 were not observed, and the abundance of NFATc1 did not change with mRspo3 treatment (Fig. 4B). These results showed that Wnt/β-catenin is the dominant Wnt signalling pathway which is enhanced by Rspo3 in the early stage of differentiation. We also confirmed that activation of the Wnt/β-catenin signalling pathway by Wnt3a increases MyHC I expression in differentiated myotubes from myoblasts derived from mixed myofibres (Supplementary Fig. 6).

### Confirmation of Wnt/β-catenin signalling contribution to Rspo3-induced MyHC I expression

**Figure 5:**
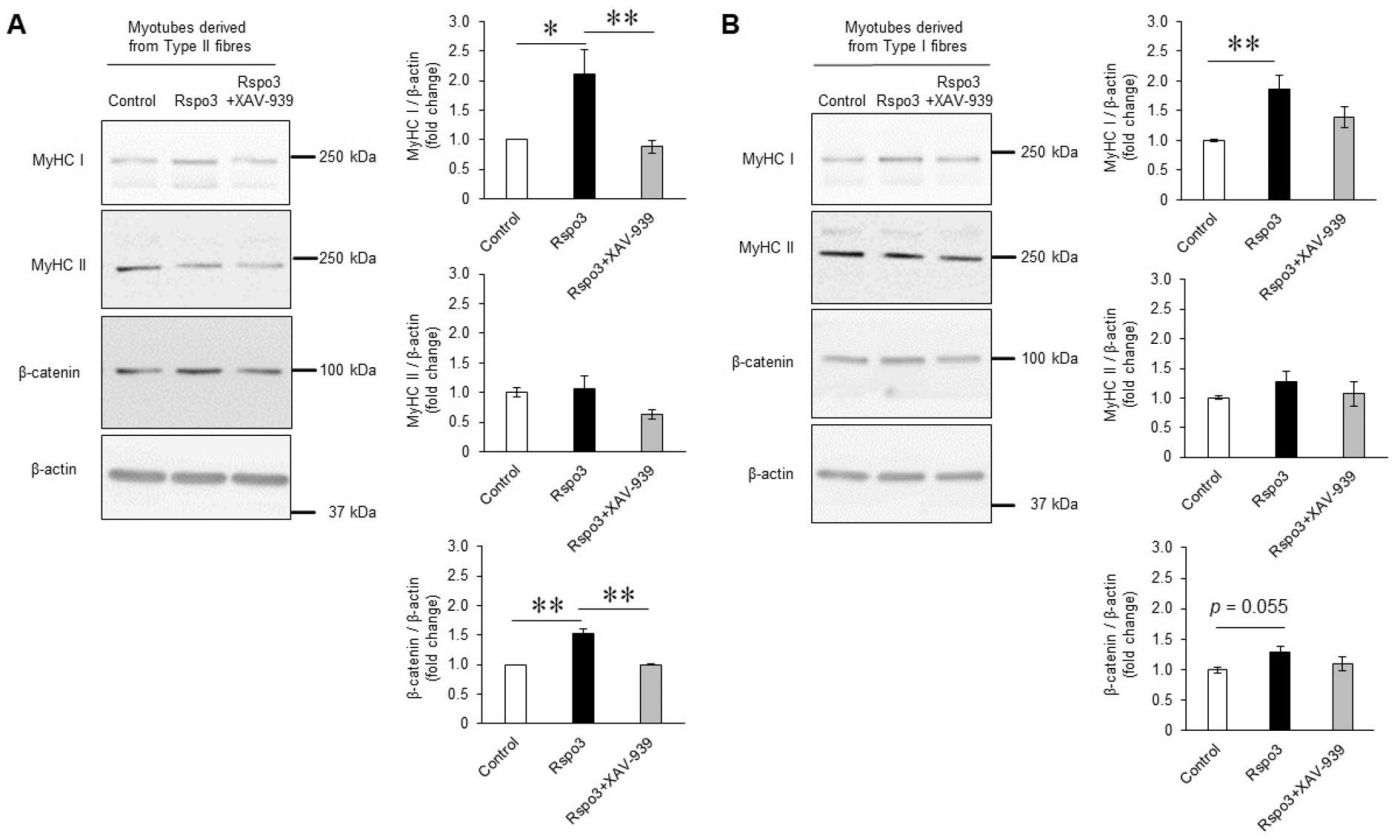
The Rspo3-induced MyHC I expression is blocked by a pharmacological inhibitor of the Wnt/β-catenin signalling pathway. (**A**, **B**) Mouse primary myoblasts were differentiated in the medium containing the Rspo3 recombinant protein (200 ng/mL; 6.45 nM) with or without the WNT/β-catenin signalling inhibitors, XAV-939 (1.56 µg/mL; 5 µM), for 3 d. Protein levels of β-catenin, MyHC I and MyHC II in myotubes were quantified using Western blotting. Values are presented as mean ± SEM (n = 6–7 mice for analysing cells derived from type II fibres, n = 9–10 mice for analysing cells derived from type I fibres). (**A**) Myoblasts derived from type II fibre were used. (**B**) Myoblasts derived from type I fibre were used. *P < 0.05; **P < 0.01 by one-way ANOVA followed by the Tukey post hoc test. Uncropped blots can be found in Supplementary Fig. 12.

To confirm that the Wnt/β-catenin signalling mediates Rspo3-induced MyHC I expression, cells were treated with XAV-939, a pharmacological inhibitor of the Wnt/β-catenin signalling pathway. Treatment of myotubes derived from type II fibres with XAV-939 during myogenic differentiation was found to significantly decrease Rspo3-induced MyHC I expression (Fig. 5A). MyHC II expression was not affected by XAV-939 treatment. This result suggests that expression of MyHC I by treatment with Rspo3 was mediated by activation of the Wnt/β-catenin signalling pathway in myotubes derived from type II fibres. On the other hand, in myotubes derived from type I fibres, though we observed a tendency for the Rspo3-induced MyHC I expression to be inhibited by XAV-939, the inhibition was not as sharp as what we saw in cells from type II (Fig. 5B). From these results, we conclude that Wnt/β-catenin is the main Wnt signalling pathway involved in the action of Rspo3. However, our data might indicate that a signalling pathway other than Wnt signalling plays a role in the Rspo3 effect in the case of type I derived myoblasts. Alternatively, myoblasts derived from type I might have lower sensitivity to the XAV-939 inhibitor than those from type II.

In this study, we found that Wnt/β-catenin signalling can serve as the main pathway to mediate the effect of Rspo3 on the increase in MyHC I expression due to the fact that the Rspo3-induced increase in MyHC I expression was returned to the control level by a pharmacological inhibitor of β-catenin, XAV-939 (Fig. 5). On the other hand, Rudolf et al. studied β-catenin knockout mice and reported that the absence of β-catenin perturbs satellite cell differentiation^27^, suggesting that the inhibition of Wnt/β-catenin signalling may be associated with the risk of a myogenic differentiation defect. To confirm this, myocytes derived from mixed myofibres (Type I + Type II) were differentiated with or without XAV-939 for three days. Differentiated myotubes were put through immunohistochemical analysis using total MyHC and Desmin antibodies to evaluate the differentiation condition of the cells (Supplementary Fig. 7). There was no observable difference between the XAV-939 non-treated control cells and the treated cells in both MyHC- and Desmin-stained images (Supplementary Fig. 7). Moreover, the fusion indices calculated from each image for the XAV-939 non-treated control cells and the treated cells were identical (Supplementary Fig. 7). These results indicate that pharmacological inhibition of the Wnt/β-catenin pathway did not impair myogenic differentiation, at least in this study protocol. The difference between Rudolf et al.’s results and ours might come from the degree of β-catenin inhibition. Inhibition realized by deletion of the β-catenin gene (i.e., knockout mice) used by Rudolf et al. could result in the absence of Wnt/β-catenin signalling as they described and be stronger than our pharmacological inhibition; in this study, XAV-939 treatment only reduced the Rspo3-induced increase in MyHC I expression compared to a control not treated with Rspo3.

## Discussion

Here, we showed that Rspo3 is specifically expressed in type I fibres, and that this myokine guides myoblasts (and satellite cells) to become MyHC I fibres through myogenic differentiation.

Previous studies indicated that β-catenin is essential for generating type I fibres during foetal limb myogenesis^33^, and that there is a possibility that the Wnt/calcium signalling pathway is required for generation of type I fibre because NFAT is known to regulate muscle fibre type^32^. In this study, Rspo3 was seen to not activate non-canonical Wnt signalling pathways during muscle differentiation; it did however activate the Wnt/β-catenin signalling pathway (Figs. 3, 4). It was also shown that a pharmacological inhibitor of the Wnt/β-catenin signalling pathway suppressed MyHC I induction, especially in myotubes derived from type II fibres (Fig. 5A). These results suggest that Wnt/β-catenin signalling is the main pathway by which MyHC I expression is induced by Rspo3. The inhibitor had a weaker effect on myoblasts derived from type I fibres compared with those from type II (Fig. 5B). This result may indicate that signalling pathways other than Wnt/β-catenin signalling are involved in the mechanism, or myotubes derived from the type I fibres have less sensitivity compared with the those from type II fibres.

The effects of Rspo3 on MyHC I upregulation are only observed when the myoblasts are treated with Rspo3 in the early stage of differentiation. Previous studies have shown that a Rspo family receptor, leucine-rich repeat-containing G-protein coupled receptor 5 (Lgr5), is rapidly upregulated in myoblasts following acute CTX induced injury^34^. Considering our results, the induction of MyHC I expression by Rspo3 during myogenesis could be activated by the Wnt/β-catenin signalling pathway via a receptor appearing in activated satellite cells, such as Lgr5.

In the present study, Rspo3 likely acted on muscle differentiation in a paracrine manner as a myokine for muscle-fibre type determination. Previous studies showed that muscle-fibre subtype determination during muscle generation is controlled by extracellular factors in muscle progenitor cells^35,36^. For example, BDNF was presumed to be a factor for type IIb fibre specification during the formation of new fibres^19^. Semaphorin 3A was a key factor in generating type I fibres in satellite cells^37^ and in generating type IIb fibres in muscle progenitor cells expressing twist family BHLH transcription factor 2^38^. Rspo3 may induce the expression of MyHC I in coordination with these factors and determine muscle fibre type during muscle generation.

A fundamental question is whether the fate of satellite cells is foreordained by the type of fibre it is located on, i.e., whether the type of fibre it differentiates to is a predetermined niche. To answer this question, previous studies prepared satellite cells from muscle tissues in which both fibre types exist^39–42^. By looking at satellite cells located on type I and II fibre types separately, we have now explicitly shown that myotubes differentiated from satellite cells from type I and type II fibres show the identical expression pattern of MyHC I and MyHC II (Fig. 2). When they are exposed to Rspo3, both types of satellite cells differentiated into myotubes which showed similarly pronounced MyHC I expression; again, we emphasize that the two populations express MyHC I and MyHC II in a similar manner (Fig. 2). At least from our results, it seems clear that the fate of satellite cells can be regulated during myogenesis. Since Rspo3 is secreted specifically from type I fibres, it may be necessary to ensure type I fibres stay as type I fibres.

Further studies are needed to unravel the roles of the Rspo3/β-catenin axis in MyHC I expression in the early stage of differentiation of myoblasts. In this study, β-catenin activation of Wnt3a also increased MyHC I expression in differentiated myotubes (Supplementary Fig. 6). This and the Rspo3 results suggest that Wnt/β-catenin signalling seems to be a necessary factor to facilitate MyHC I expression. Recently, it has been reported that Lgr5, a receptor for the R-spondin family and a potent mediator of Wnt/β-catenin signalling, is upregulated in satellite cells upon muscle injury, contributing to muscle regeneration and replenishment of the satellite cell pool^34^. As Rspo3 is the dominant member expressed in myofibre (Supplementary Fig. 2), its role in homeostasis and plasticity of satellite cells should be a topic for future inquiry.

In the present study, we showed the paracrine function of Rspo3 for muscle fibre type determination using an in vitro experiment. In general, Rspo3 is recognised ubiquitously as being expressed throughout the developing body for proper organ formation^43–45^. Furthermore, Rspo3 is required for maintaining gastric epithelial stem cells in response to *H. pylori* infection^46^, for epithelial regeneration in the colon^47^, and for preventing the formation of anti-inflammatory interstitial macrophages^48^. To further elucidate the function of Rspo3 as a myokine, future studies should focus on Rspo3 derived from adult muscle fibres.

## Methods

### Animals

Adult (8–16-week old) male transgenic mice expressing CFP under the control of the Myh7 promoter were used in this study^20^. The mice were obtained from the Jackson Laboratory (Myh7-CFP mouse, Stock no. 016922), housed at 23–25 °C with a 12 h light/dark cycle, and received a normal chow diet and water ad libitum. All experiments protocols and care of the laboratory animals were approved by the Guidelines of the Experimental Animal Committee of Tokyo Metropolitan University and followed the Guidelines for the Proper Conduct of Animal Experiments, as established by the Science Council of Japan (A3-6, A3-7, A3-9). This study was conducted in compliance with the ARRIVE guidelines.

### Isolation of single muscle fibres and primary cell culture

SOL was isolated from Myh7-CFP mice, and single muscle fibres were digested using type I collagenase. To collect muscle fibres for RNA isolation and real-time quantitative PCR analysis, the muscles were incubated with 4% (w/v) collagenase for 1 h at 37 °C. For culturing satellite cells, the collagenase concentration was 0.8% (w/v) and the incubation time was 2 h at 37 °C. Single muscle fibres were collected under a stereomicroscope; cells and debris other than muscle fibres were removed. CFP fluorescence was then observed under a fluorescence stereomicroscope (Leica M165 FC), and each muscle fibre was identified as type I (CFP-positive fibre) or type II (CFP-negative fibre).

Satellite cells were cultured according to our previous report^49^ but with some modifications. Briefly, each type of muscle fibre was pooled and incubated with Accutase (Innovative Cell Technologies, SAN, USA) for 10 min at room temperature and cultured on Matrigel-coated dishes. Satellite cells were cultured in growth medium (No glucose DMEM supplemented with 30%(v/v) FBS (NICHIREI BIOSCIENCES INC., Tokyo, Japan), 1%(v/v) GlutaMAX, 1%(v/v) chicken embryo extract, 10 ng/mL bFGF and 1%(v/v) penicillin–streptomycin) at 37 °C with 5% CO_2_. Myoblasts derived from satellite cells were reseeded onto 12 well plates coated with Matrigel (Corning, NY, USA) at a density of 3.1 × 10^4^ cells/cm^2^ with 2 mL of growth medium. One day after seeding, the medium was switched to differentiation medium (DMEM GlutaMAX supplemented with 5% horse serum (Thermo Fisher Scientific, MA, USA) and 1% penicillin–streptomycin).

To analyse the role of Rspo3 in vitro, recombinant mouse Rspo3 protein (R&D Systems, MN, USA) was added to the differentiation medium at a final concentration of 100, 200, and 400 ng/mL (3.225, 6.45, 12.9 nM); a medium containing BSA at a final concentration of 200 ng/mL was used as a control culture. For the Wnt/β-catenin signalling inhibition experiment, XAV-939 (Chem Scene LLC, NJ, USA) was added to the differentiation medium at a final concentration of 1.56 µg/mL (5 µM). For the experiment looking at pharmacological stimulation of Wnt/β-catenin signalling, Wnt3a (R&D Systems) was added to the differentiation medium at a final concentration of 40 ng/mL (1.07 nM), and a medium containing BSA at a final concentration of 40 ng/mL was used as a control culture. The differentiation medium was changed daily. The cells were cultured for three or six days and used for subsequent experiments.

### RNA isolation and real-time quantitative PCR analysis

Total RNA was extracted from each muscle fibre type and tissues (SOL, TA, EDL, ovary, lung) using Trizol reagent (Invitrogen, CA, USA). The presence of Rspo3 transcript was analysed by conventional PCR (RT-PCR). PCR was performed using TaKaRa Taq (TaKaRa, Shiga, Japan) with specific primer pairs. Primer sequences for this study can be found in Supplementary Table 1. Quantitative real-time PCR (qRT-PCR) was performed on a 96-well PikoReal Real-Time PCR System with a DyNAmo ColorFlash SYBR Green qPCR Kit (Thermo Fisher Scientific, MA, USA) according to the manufacturer’s protocol. The mRNA levels of each gene were normalised to those of the housekeeping gene GAPDH and TATA binding protein (Tbp). Primer sequences for this study can be found in Supplementary Table 2. The mRNA content of both Rspo3 and Tbp was calculated from the cycle threshold values using a standard curve, and the ratio between Rspo3 and Tbp was calculated. Primers were synthesised by Eurofins Genomics Co., Ltd. (Tokyo, Japan).

### Western blotting

Immunoblotting was performed as described previously^50^. Total protein extracts were obtained from homogenised tissues and cultured cells, and lysed with lysis buffer. To fractionate the cell lysate into cytoplasmic and nuclear fractions, the NE-PER Nuclear and Cytoplasmic Extraction Reagents Kit (Thermo Fisher Scientific) was used. Protein concentrations of all samples were measured using a Bradford protein assay (Bio-Rad, CA, USA). Tissue and cell lysates were separated by sodium dodecyl sulfate-poly-acrylamide gel electrophoresis (SDS-PAGE) and transferred to polyvinylidene fluoride membranes (PVDF). After membranes were cut for blocking with an appropriate blocking reagent, the membranes were blocked with tris-buffered saline containing 0.1% Tween 20, 5% non-fat dry milk (for proteins except NFATc1, Phospho-SAPK (Stress-activated protein kinase)/JNK and SAPK/JNK), 5% bovine serum albumin (for NFATc1), or 2.5% non-fat dry milk and 2.5% bovine serum albumin (for Phospho-SAPK/JNK and SAPK/JNK). Membranes were then incubated overnight with appropriate primary antibodies (Supplementary Table 3). Secondary antibodies conjugated to horseradish peroxidase (GE Healthcare, Buckinghamshire, UK) were used for detection with enhanced chemiluminescence (PerkinElmer, MA, USA). The Western blotting data was found using ImageQuant TL Image Analysis Software (Cytiva, MA, USA).

### Immunohistochemistry and fluorescence imaging of isolated single fibres

Mouse SOL was frozen in liquid nitrogen-cooled isopentane in a Tissue-Tek OCT (Sakura Finetek Japan Co., Ltd., Tokyo, Japan). 10-μm sections were cut using a cryostat (CM 1950; Leica Micro systems, Wetzlar, Germany). The serial sections were mounted onto APS-coated slides (Matsunami, Osaka, Japan) for histochemical staining. Muscle sections were fixed with 4% paraformaldehyde (PFA) for 10 min at room temperature (23–25 °C). Slides were washed with phosphate buffered saline (PBS), incubated for 30 min in PBS containing 0.3% Tween 20, washed in PBS, incubated for 30 min in 5% goat serum in PBS then overnight at 4 °C in the primary antibody, washed in PBS, incubated for 1 h at room temperature (23–25 °C) in the secondary antibody, washed in PBS, and mounted with VECTASHIELD mounting media (H-1000) (VECTOR LABORATORIES, INC., CA, USA). Antibodies for this study can be found in Supplementary Table 3.

For fluorescence imaging of isolated single fibres, collected single fibres were fixed with 4% PFA followed by mounting with VECTASHIELD mounting media with DAPI (H-1200) (VECTOR LABORATORIES, INC.).

Images were captured using a Nikon ECLIPSE Ti, a Plan Fluor 20X objective lens, and NIS-Elements Image Analysis Software (Nikon, Tokyo, Japan). The LUT is linear and covers the full range of the data.

### Immunocytochemistry of myotubes

Myotubes were washed with PBS and immediately fixed with 4% PFA for 10 min at room temperature (23–25 °C). Fixed cells were washed with PBS, incubated for 30 min in 10% goat serum in PBS containing 0.3% Tween 20 at room temperature (23–25 °C), incubated overnight at 4 °C in the primary antibody in PBS containing 0.025% Tween 20, and washed in PBS. They were then incubated for 1 h at room temperature (23–25 °C) in the secondary antibody in PBS containing 0.025% Tween 20, and washed in PBS. The nuclei were then counterstained with DAPI in PBS. Antibodies for this study can be found in Supplementary Table 3. We also analysed the fusion index, which represents the percentage of MyHC-positive or desmin-positive nuclei among the total nuclei within each field, using KEYENCE BZ-X800, a Plan Apochromat 10X objective lenses, and KEYENCE BZ-X series Image Analysis Software (KEYENCE, Osaka, Japan). The LUT is linear and covers the full range of the data. The resolution of the image was compressed for KEYENCE BZ-X series Image Analysis Software when images were analysed. 107 images per well were used for the fusion index analysis.

### Statistical analyses

All values are shown as the mean ± standard error of the mean (SEM). Two-tailed unpaired Student’s t-tests were used to compare groups. For multiple comparisons, data were analysed using a one-way ANOVA followed by the Tukey post hoc test. The level of significance was set to *P* < 0.05.

## Supplementary Information

Supplementary Information 1.

Supplementary Information 2.

## Data Availability

All data presented in this study are available in the main text, the main figures, or the supplementary materials. Source data are provided with this paper.
