# Supplementary material for: R-spondin3 is a myokine that differentiates myoblasts to type I fibres
Source: Sci Rep. 2022 Jul 29;12:13020. doi: 10.1038/s41598-022-16640-2 (PMC9338073; doi:10.1038/s41598-022-16640-2)
Supplement: Supplementary file 2 — Supplementary Information 2. [file 41598_2022_16640_MOESM2_ESM.pdf]

Supplementary Information

R-spondin3 is a myokine that differentiates myoblasts to type I fibres

Yoshitaka Mita[1], Haonan Zhu[1], Yasuro Furuichi[1], Hiroki Hamaguchi[1], Yasuko Manabe[1], Nobuharu L. Fujii[1,*]

[1] *Department of Health Promotion Sciences, Graduate School of Human Health Sciences, Tokyo Metropolitan University, Tokyo, Japan.*

[*]*Corresponding Author*

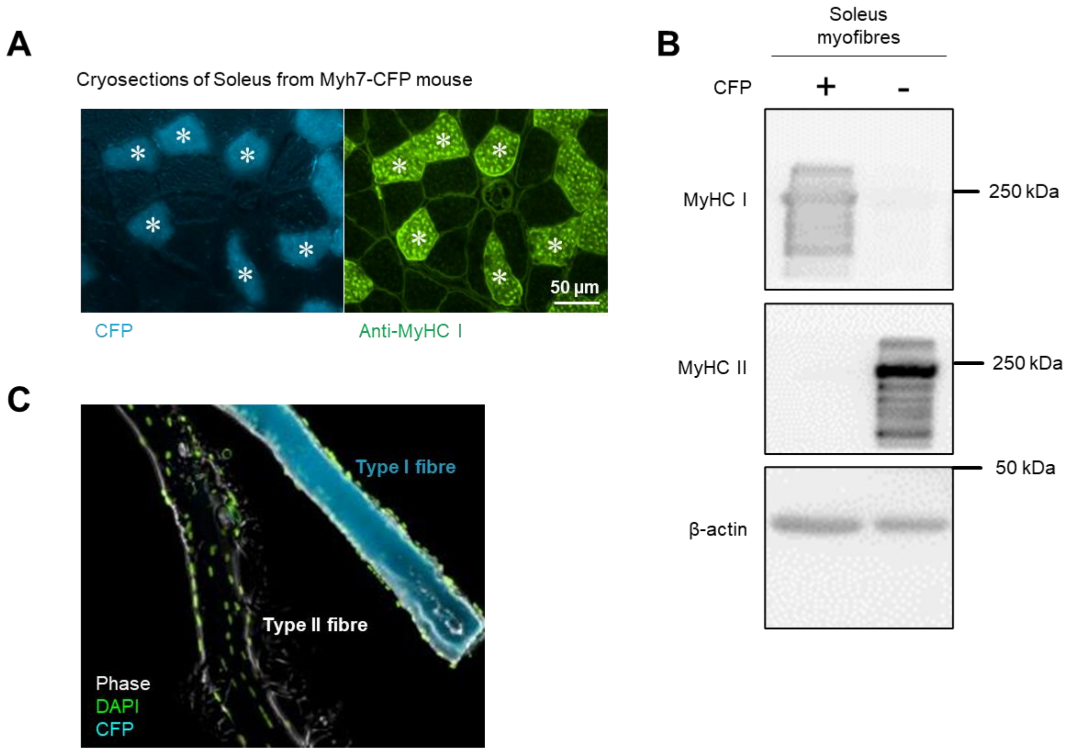

Supplementary Figure 1. CFP is selectively expressed in Type I fibres derived from the Myh7-CFP mouse.

(A) Representative cryosections of soleus muscles from a Myh7-CFP mouse. CFP was expressed specifically in MyHC I positive fibres. * indicates identical fibres. (B) Collected CFP positive muscle fibres specifically expressed MyHC I protein but not MyHC II. (C) Muscle fibres isolated from the soleus were observed under a stereomicroscope. Uncropped blots can be found in Supplementary Figure 13.

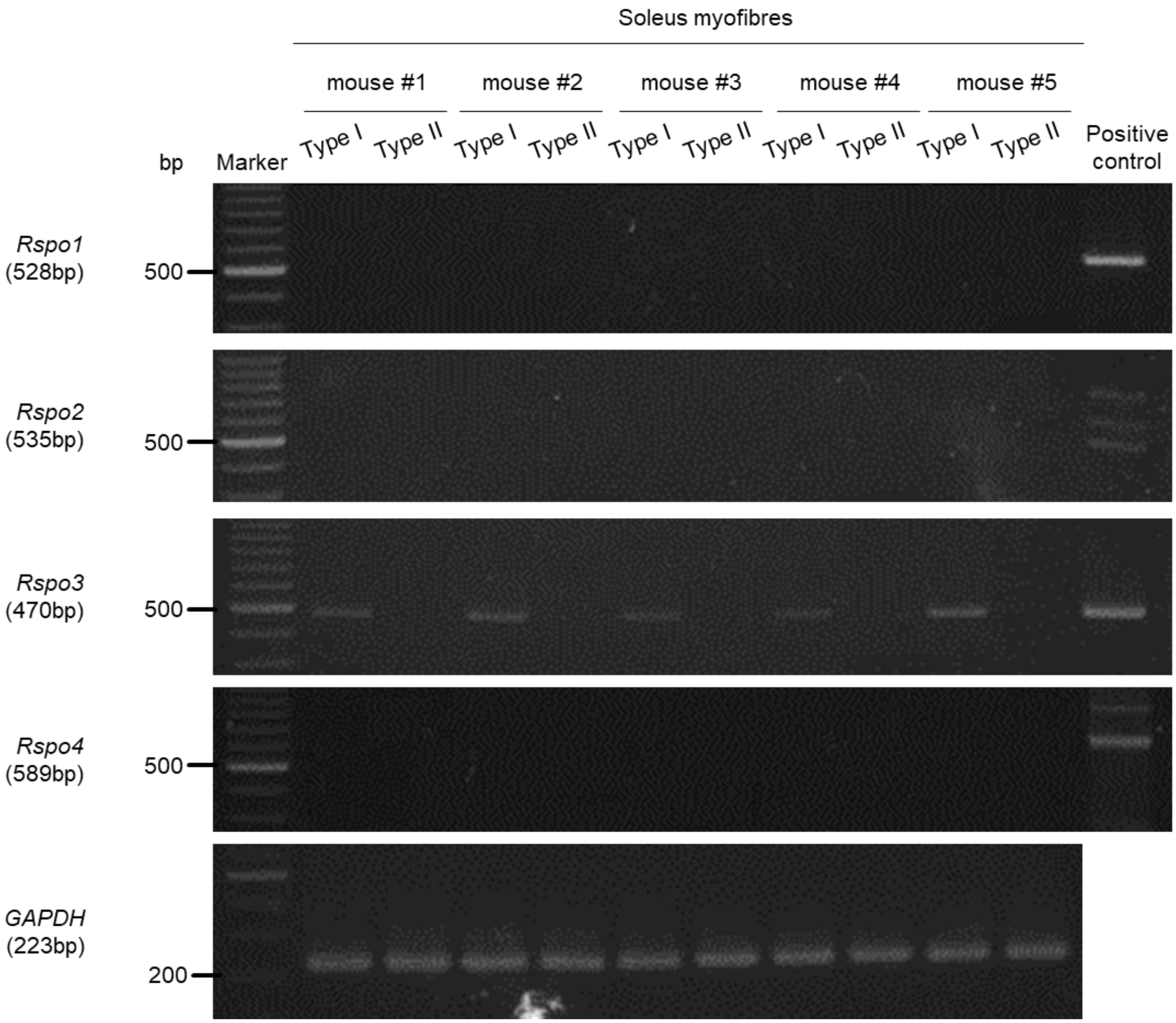

Supplementary Figure 2. Analysis of R-spondin family member expression levels in murine skeletal muscle fibres.

Gene expression of R-spondin family members were analysed in type I and II fibres (n = 5) using conventional RT-PCR. Positive controls for *Rspo1*, *Rspo2* and *Rspo3* were RT-PCR products; mRNAs were extracted from the ovary. For *Rspo4*, mRNAs were extracted from the lung. Uncropped gels can be found in Supplementary Figure 14.

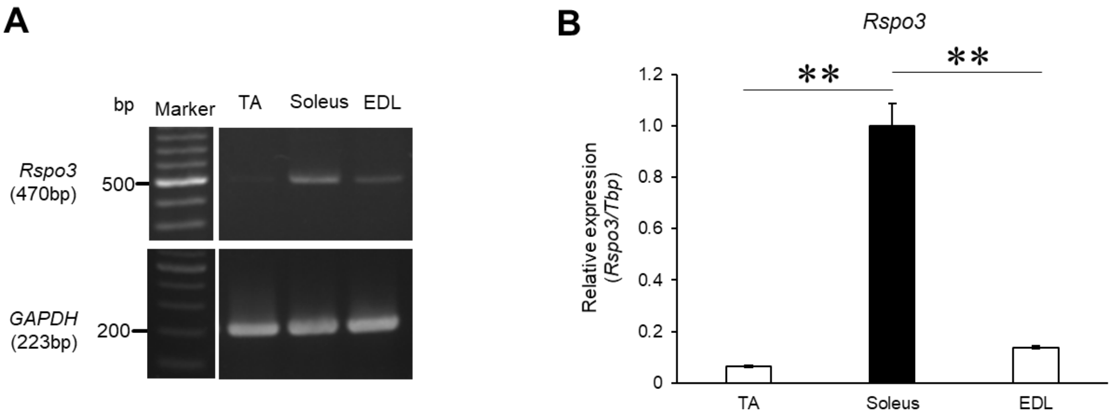

Supplementary Figure 3. Analysis of the *Rspo3* expression level in murine skeletal muscle tissue.

(A) The mRNA expression of the *Rspo3* gene was analysed in mouse muscle tissues using conventional RT-PCR (n = 3 mice). TA; Tibialis anterior, EDL; extensor digitorum longus. (B) The mRNA expression level of *Rspo3* in mouse muscle tissues was measured by quantitative RT-PCR analysis (n = 6 mice). Values are presented as mean ± SEM. **$p < 0.01$ by one-way ANOVA followed by the Tukey post hoc test. Uncropped gels can be found in Supplementary Figure 15.

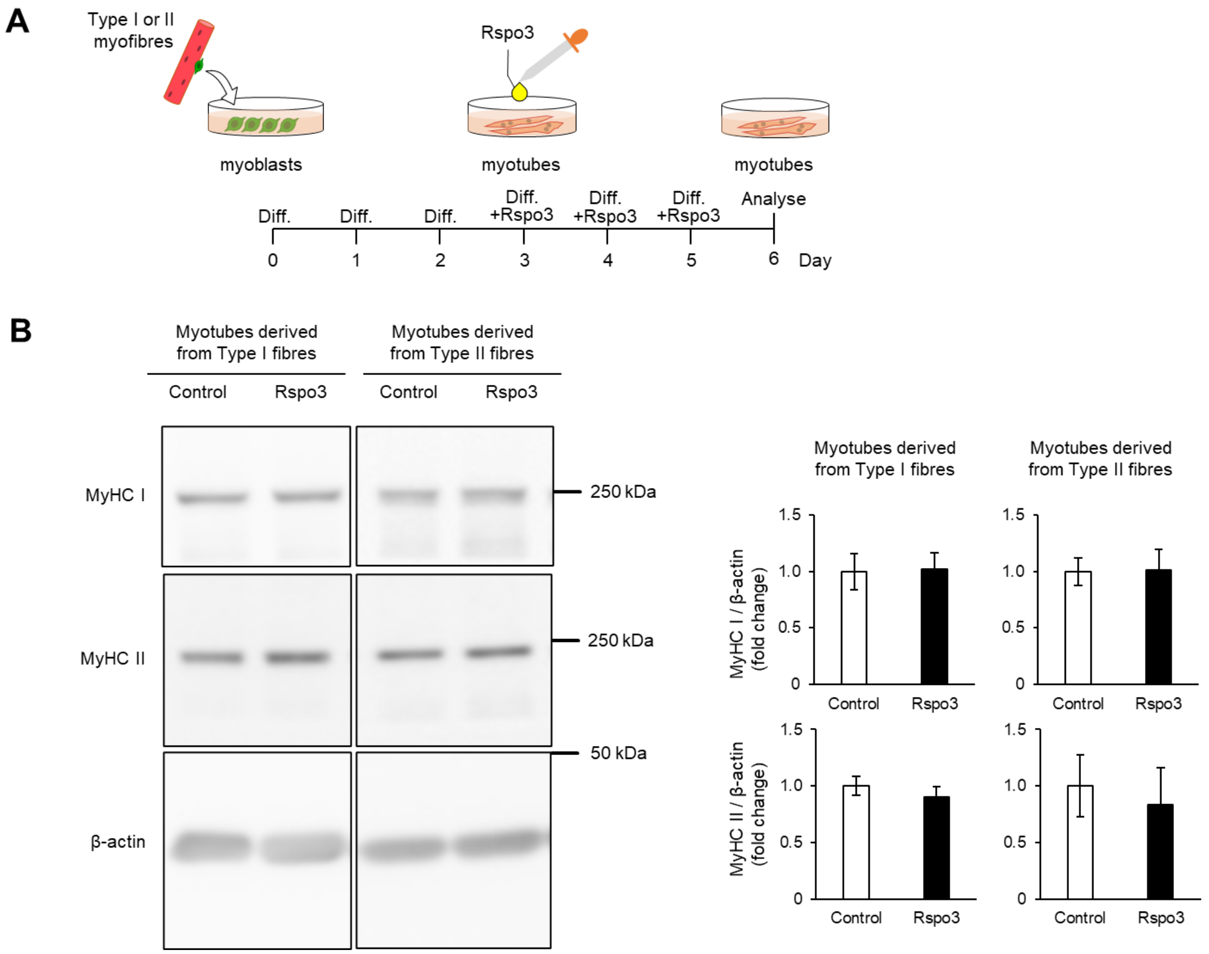

Supplementary Figure 4. Treatment of myotubes with Rspo3 protein after differentiation does not induce expression of MyHC I.

(A) Experimental scheme. Mouse primary myotubes derived from satellite cells of type I and type II muscle fibres were cultured for 3 d in a differentiation medium containing BSA (200 ng/mL; Control) or mouse Rspo3 recombinant protein (200 ng/mL; 6.45 nM). (B) Protein levels of MyHC I and MyHC II in myotubes were analysed by Western blotting. Values are presented as mean ± SEM (n = 6 mice). Uncropped blots can be found in Supplementary Figure 16.

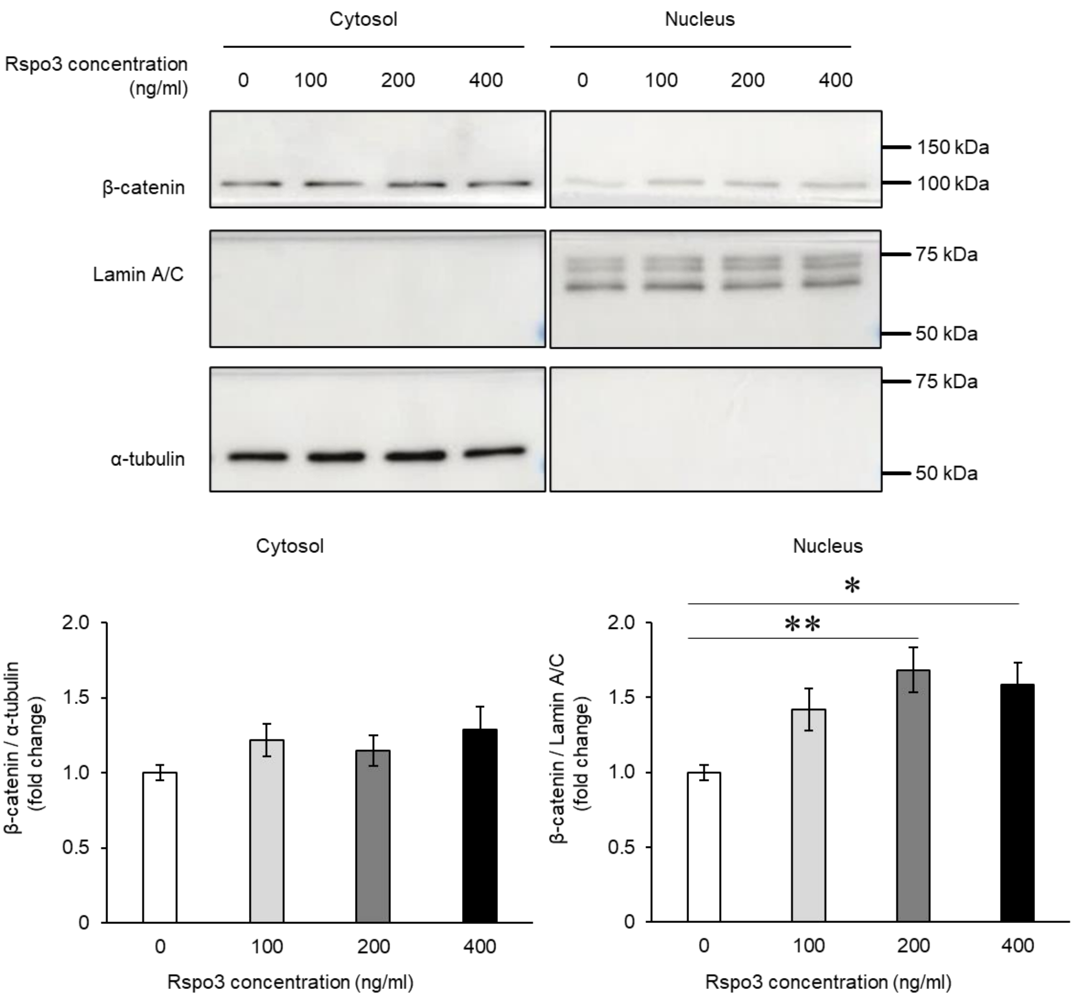

Supplementary Figure 5. The dose responsiveness of β-catenin to treatment of myotubes with Rspo3 protein in cytoplasmic and nucleic fractions.

(A, B) Mouse primary myoblasts derived from satellite cells of soleus were differentiated for 3 d in the absence (BSA; 200 ng/mL) or presence of the mouse Rspo3 recombinant protein (100, 200, 400 ng/mL; 3.225, 6.45, 12.9 nM). Cytoplasmic and nucleic fractions were extracted from myotube lysate. Protein levels of β-catenin, lamin A/C (nuclear maker), and α-tubulin (cytosol maker) in myotubes were analysed using Western blotting. Values are presented as mean ± SEM (n = 16). *$p < 0.05$; **$p < 0.01$ by one-way ANOVA followed by the Tukey post hoc test. Uncropped blots can be found in Supplementary Fig. 17.

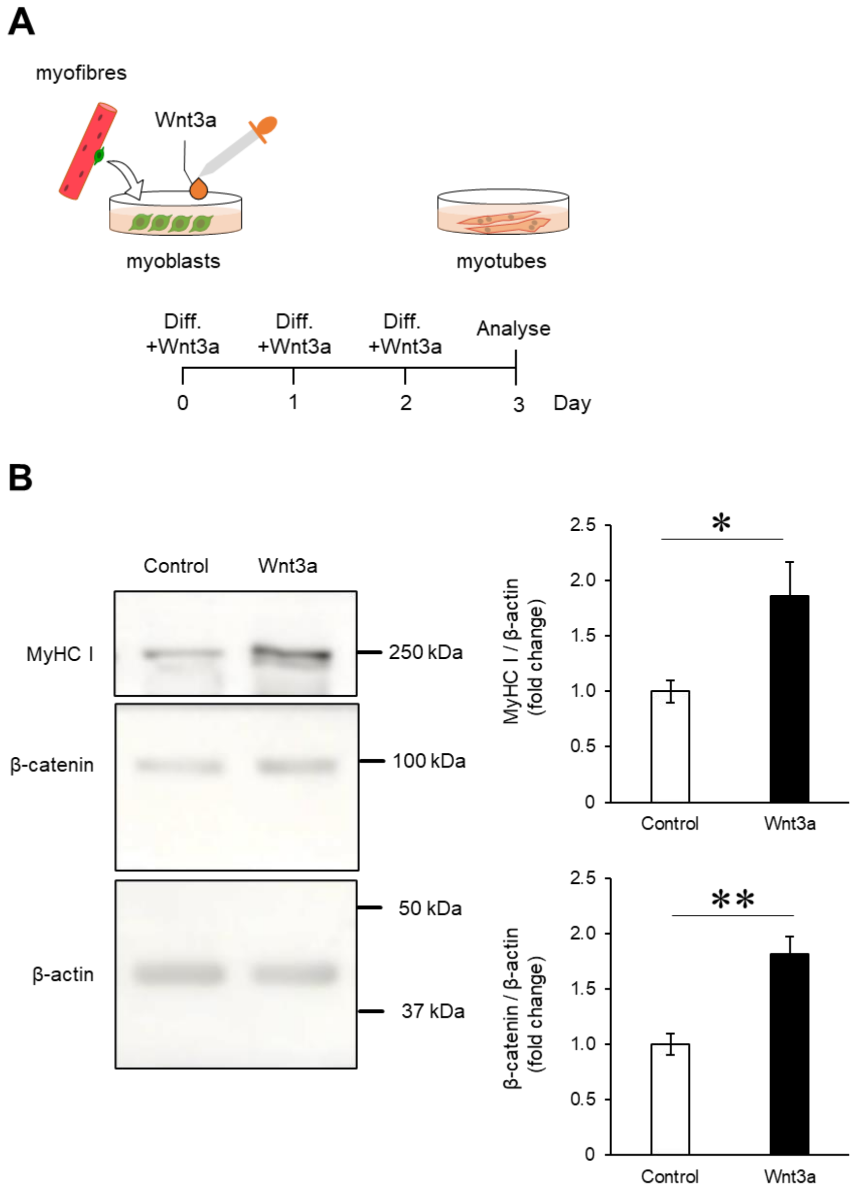

Supplementary Figure 6. Stimulation of Wnt/β-Catenin signalling increases MyHC I expression in myotubes.

(A) Experimental scheme. Mouse primary myotubes derived from satellite cells were cultured for 3 d in a differentiation medium with or without Wnt3a (40 ng/mL; 1.07 nM). (B) Protein levels of β-catenin and MyHC I in the differentiated myotubes were measured by Western blotting. The expression of these proteins was normalised to that of β-actin. Values are presented as mean ± SEM (n = 9 mice). *p < 0.05; **p < 0.01 by Student's t-test. Uncropped blots can be found in Supplementary Figure 18.

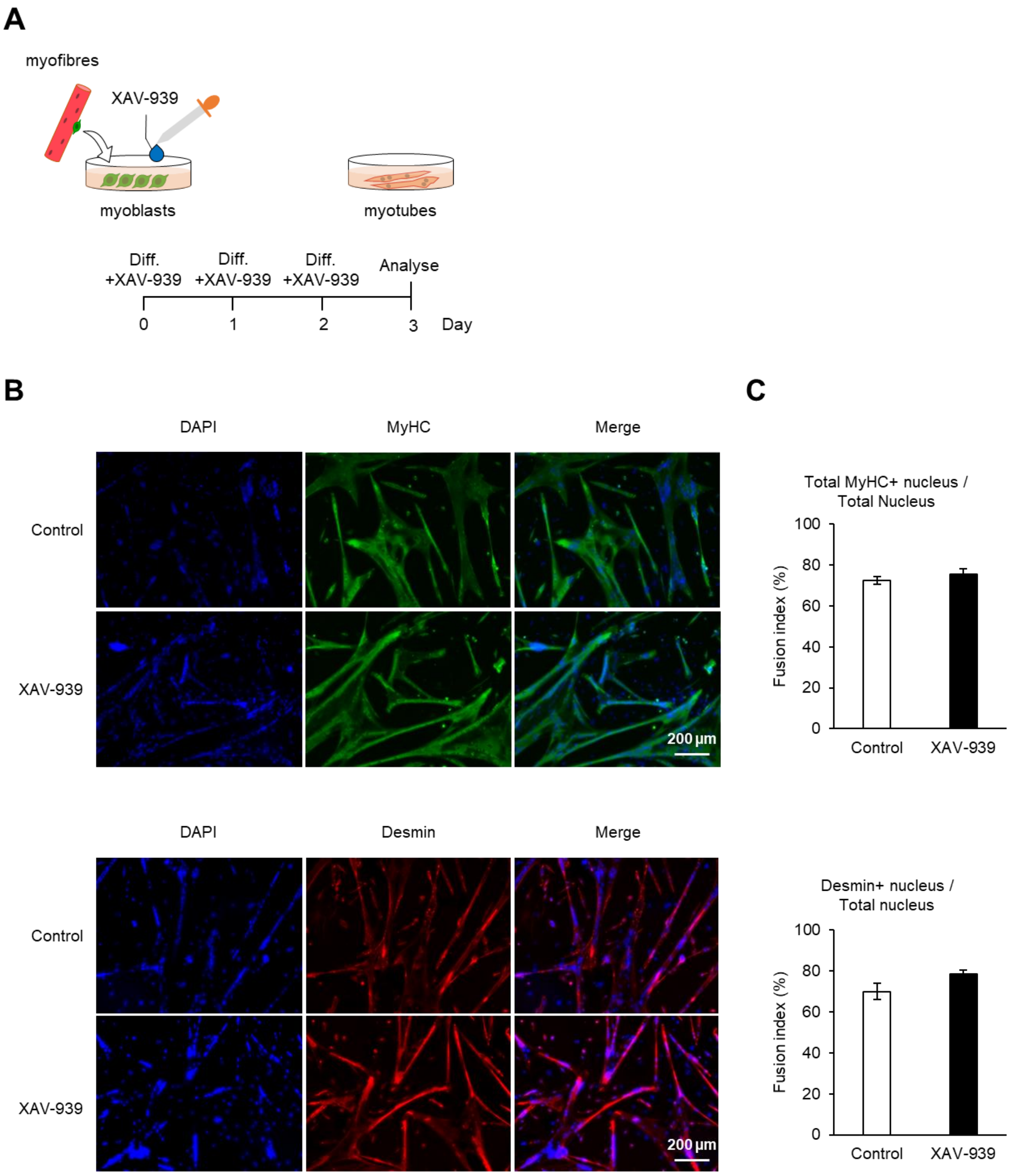

Supplementary Figure 7. β-catenin inhibition does not affect progression of myogenic differentiation.

(A) Experimental scheme. Mouse primary myotubes derived from satellite cells were cultured for 3 d in a differentiation medium with or without XAV-939 (1.56 µg/mL; 5 µM). (B) Myotubes were stained with antibodies to total myosin heavy chain (MyHC) (green) and desmin (red). Nuclei were counterstained with DAPI (blue). (C) Fusion index of MyHC positive nuclei or desmin. XAV-939 is the β-catenin inhibitor.

Fig. 1A

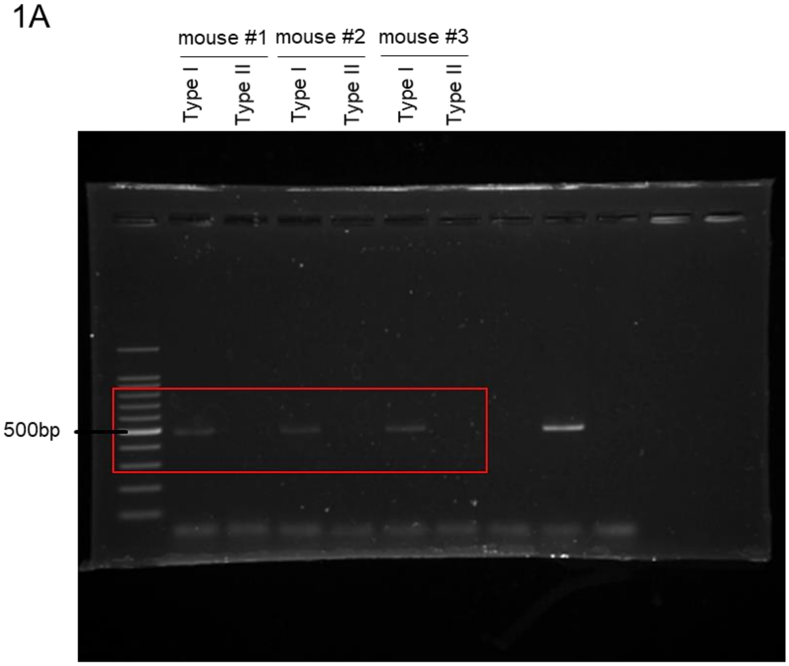

Rspo3

Fig. 1A

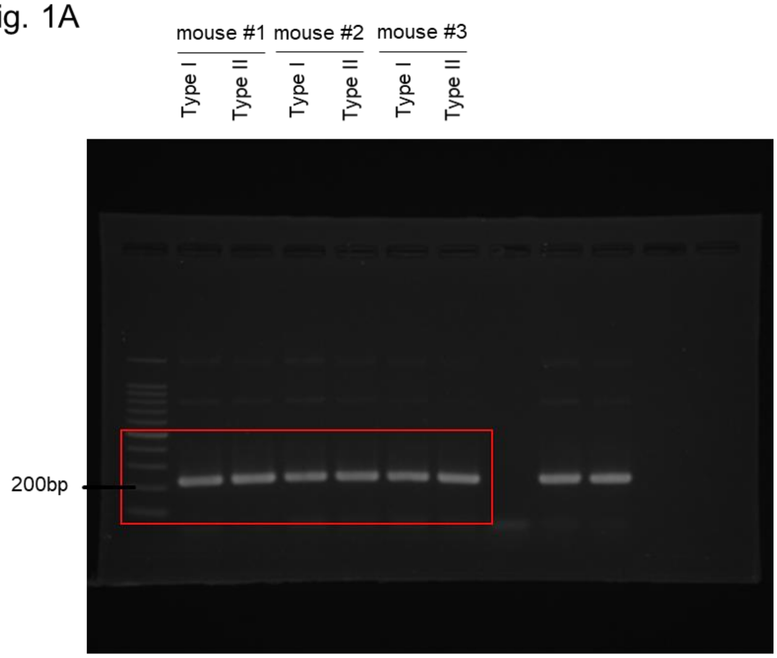

GAPDH

Supplementary Figure 8. Uncropped images of gel panels in Figure 1.

The cropped images are outlined in red.

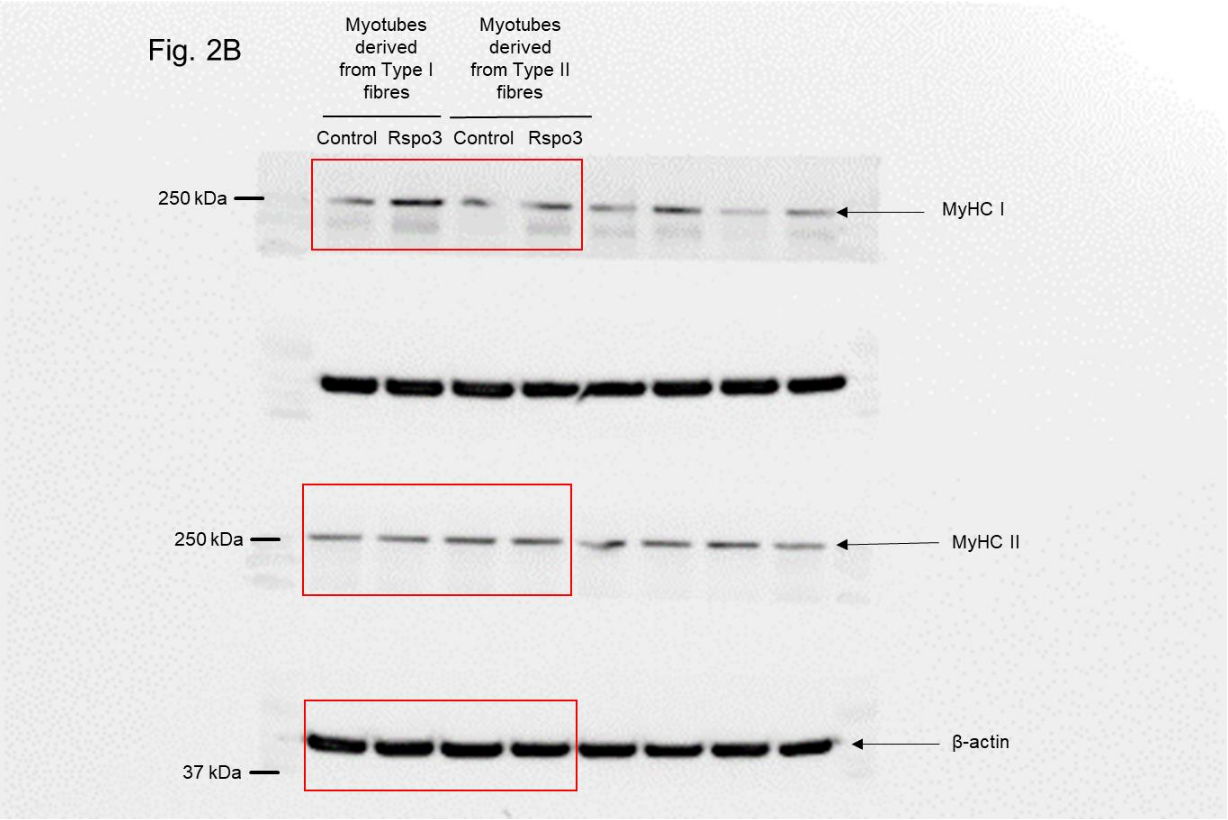

Supplementary Figure 9. Uncropped images of Western blot in Figure 2.

The cropped images are outlined in red.

Fig. 3A

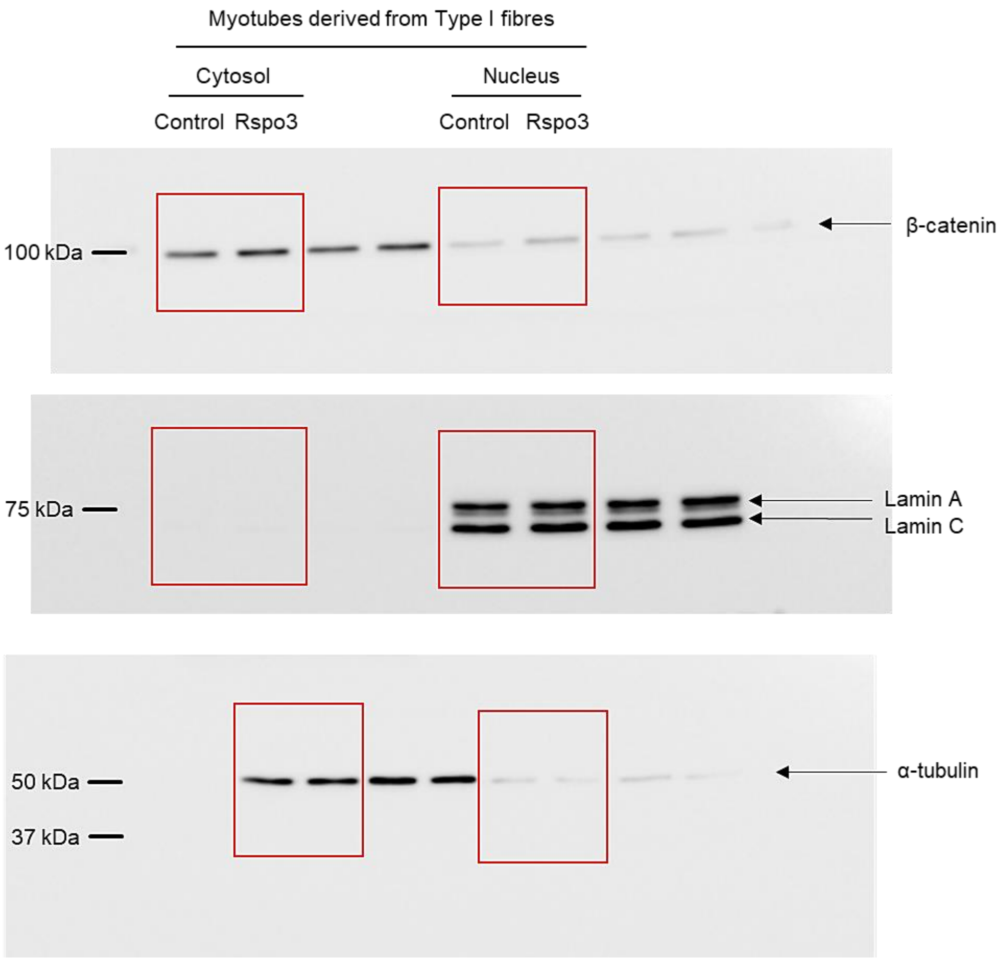

 (*legend on next page*)

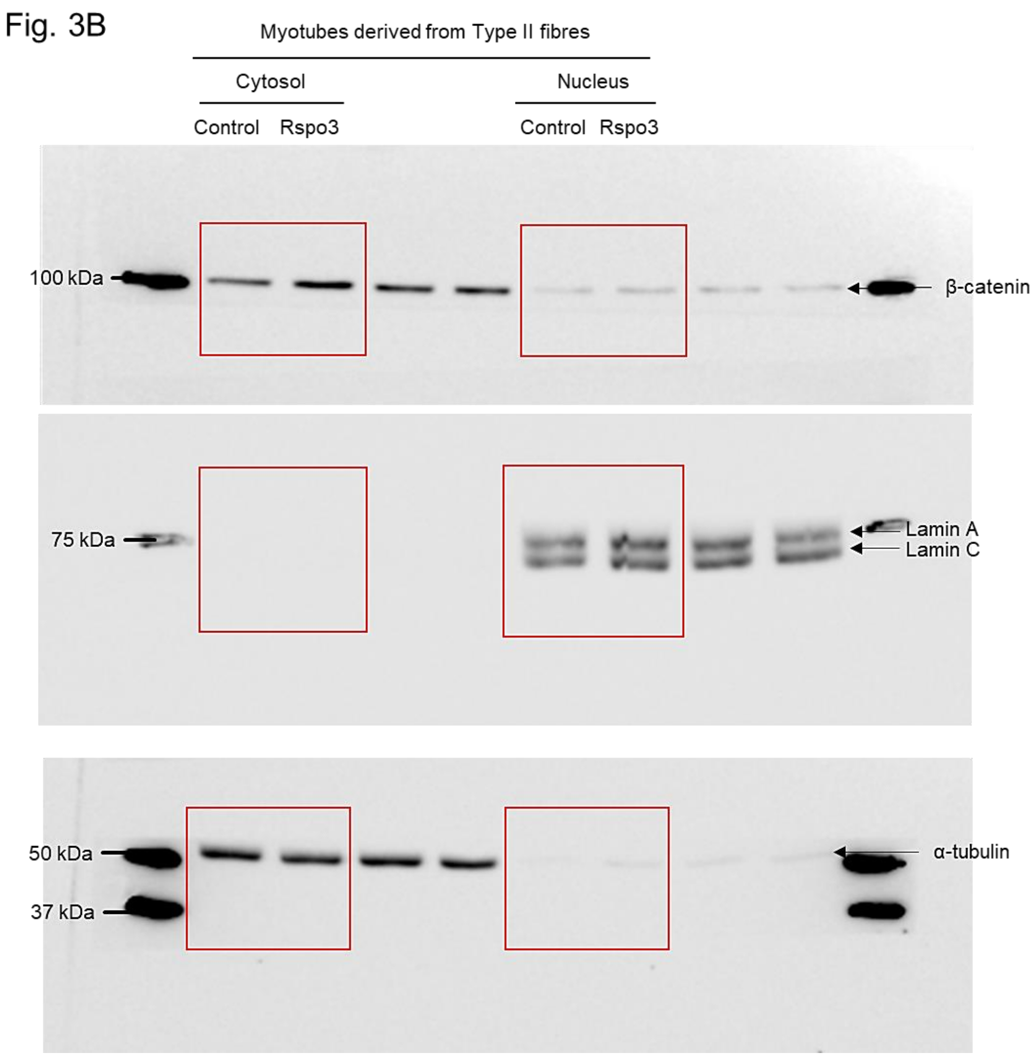

Supplementary Figure 10. Uncropped images of Western blot in Figure 3.

The cropped images are outlined in red.

Fig. 4A

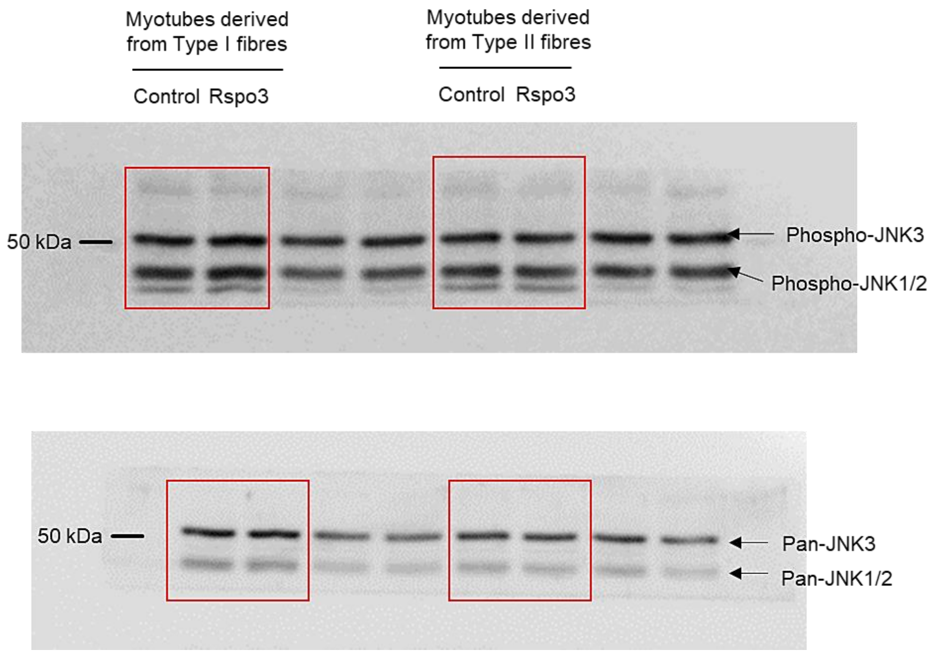

Fig. 4B

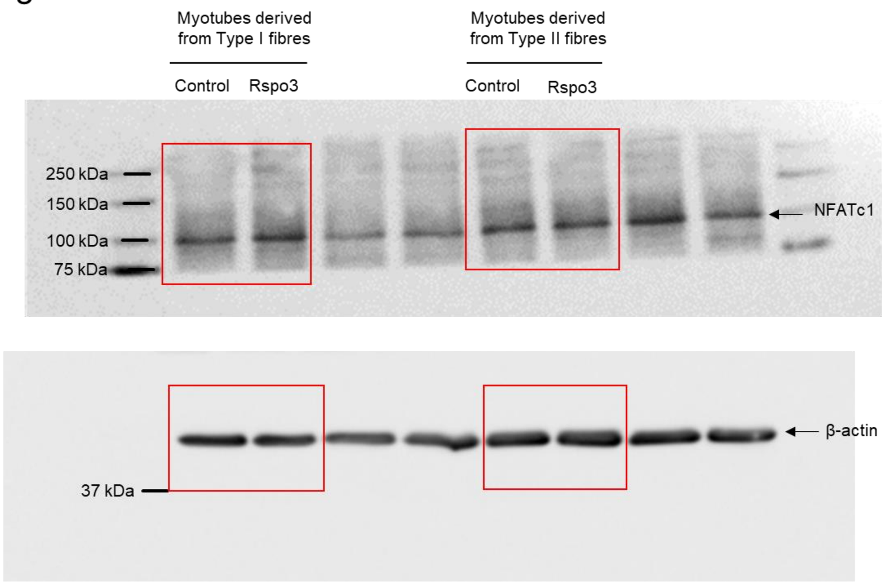

Supplementary Figure 11. Uncropped images of Western blot in Figure 4.

The cropped images are outlined in red.

Fig. 5A

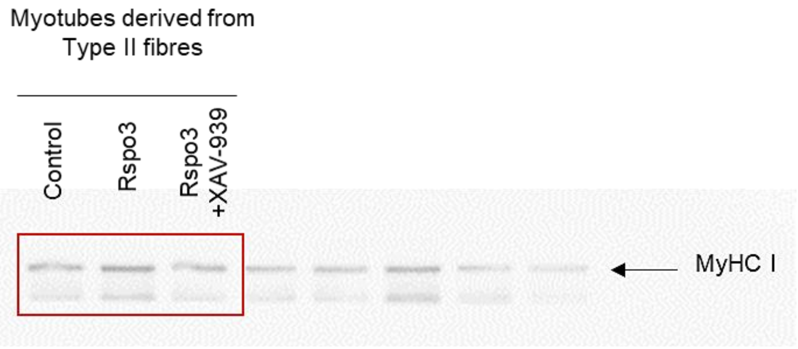

Fig. 5B

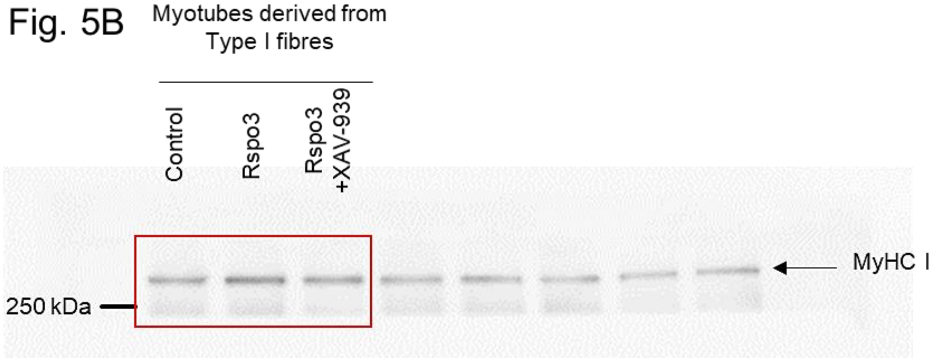

Fig. 5B

Fig. 5A

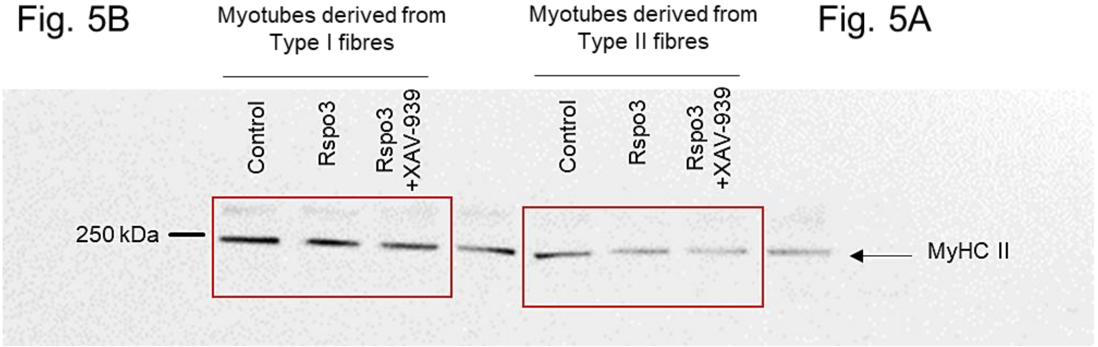

Fig. 5A

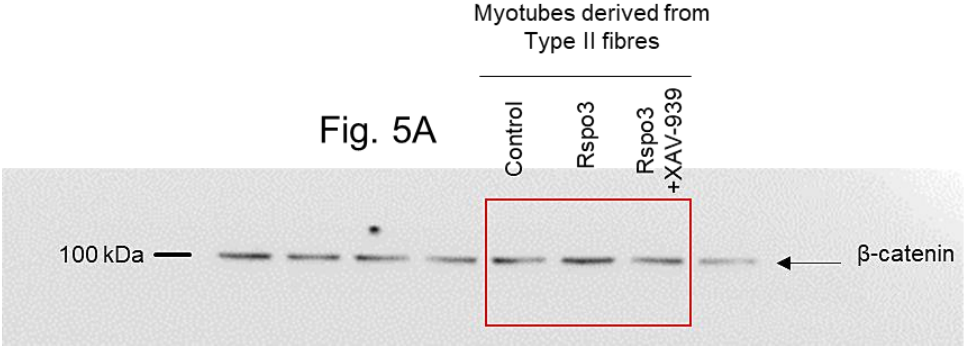

(*legend on next page*)

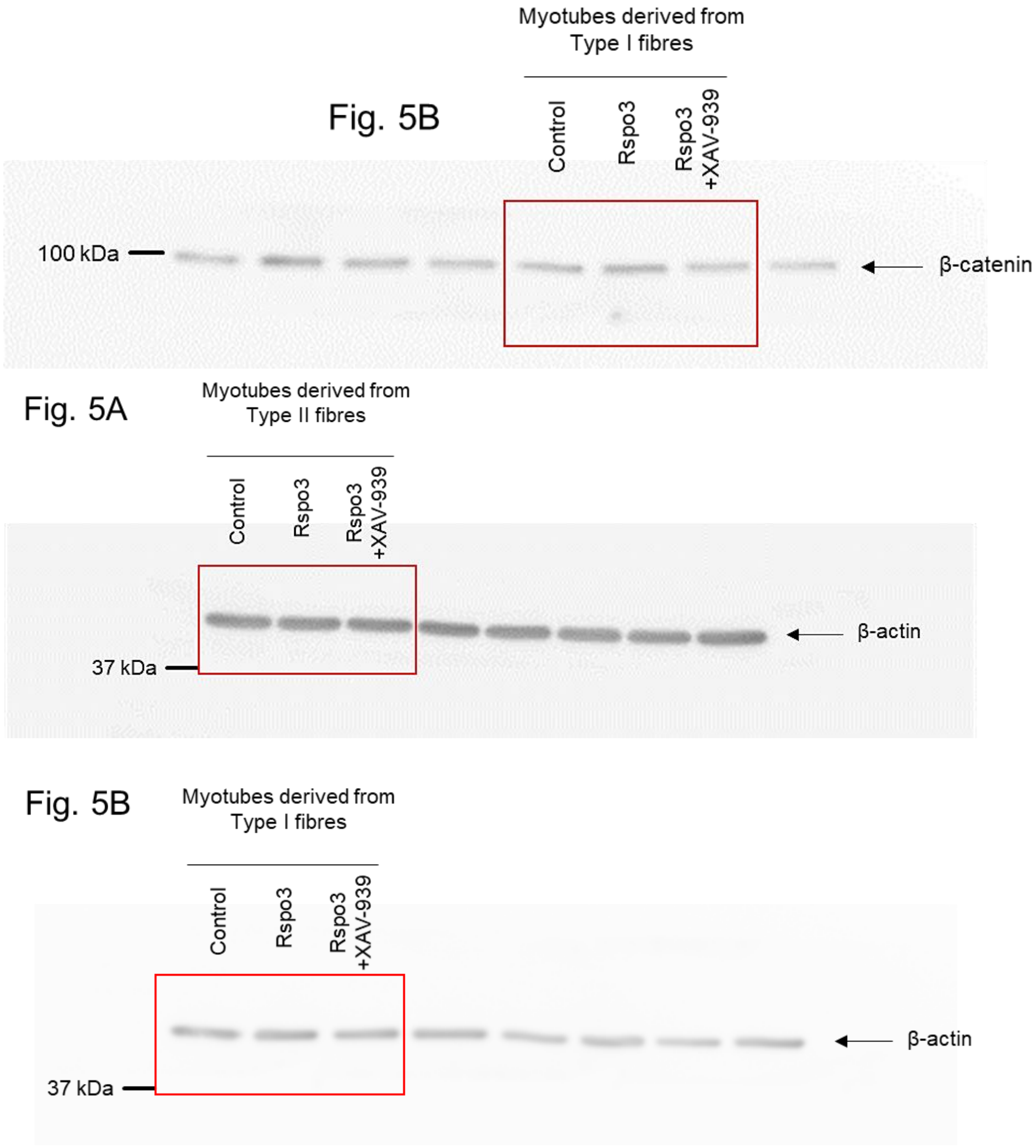

Supplementary Figure 12. Uncropped images of Western blot in Figure 5.

The cropped images are outlined in red.

Supplementary Fig. 1B

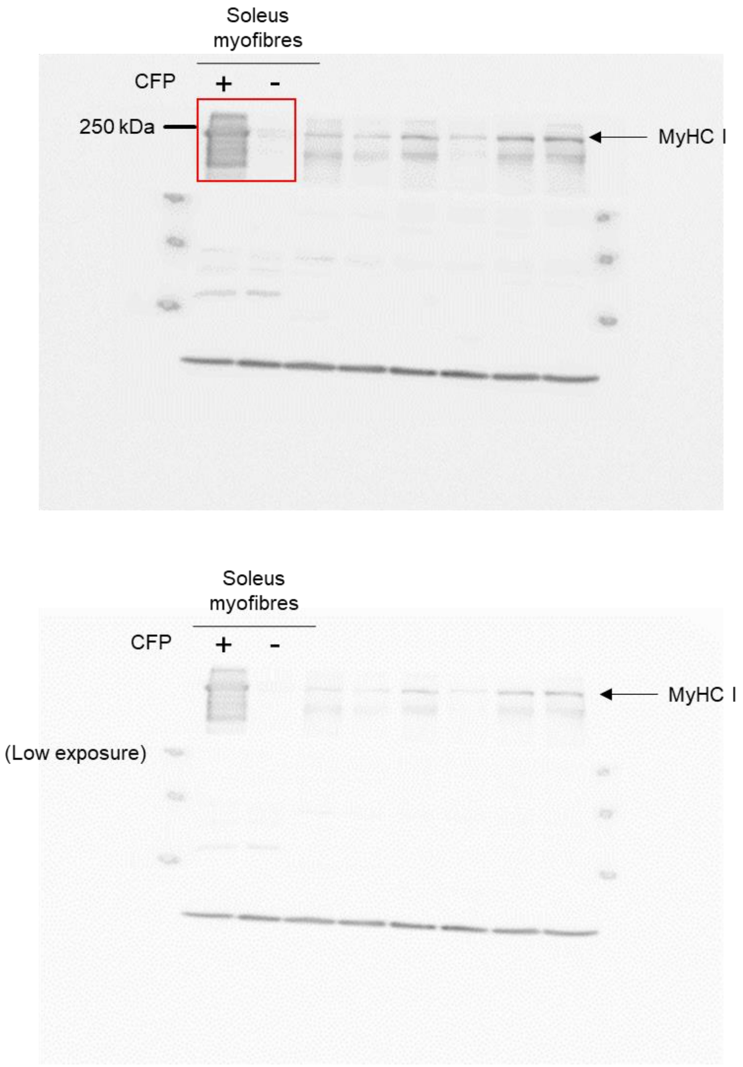

(*legend on next page*)

Supplementary Fig. 1B

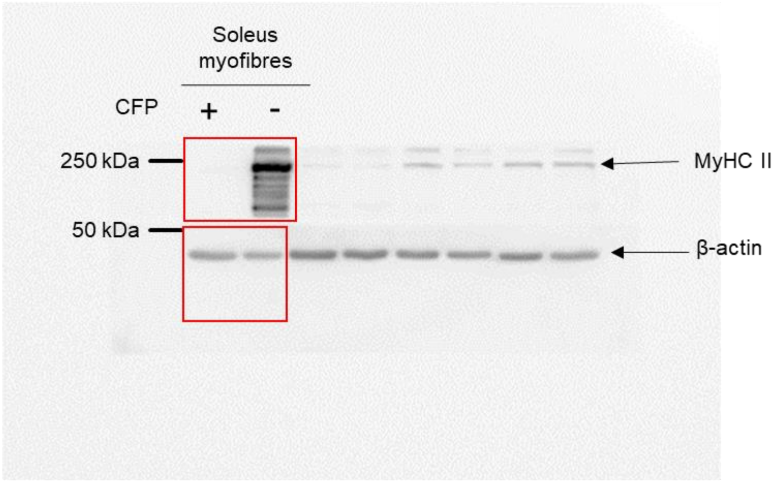

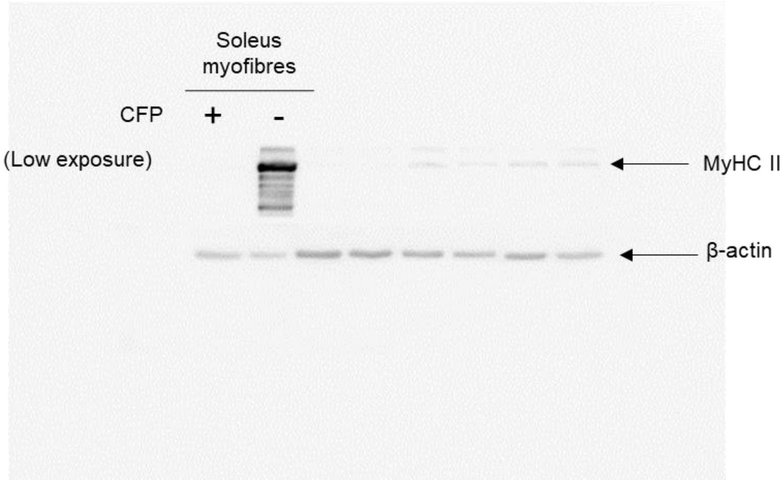

Supplementary Figure 13. Uncropped images of Western blot in Supplementary Figure 1.

The cropped images are outlined in red.

Supplementary Fig. 2

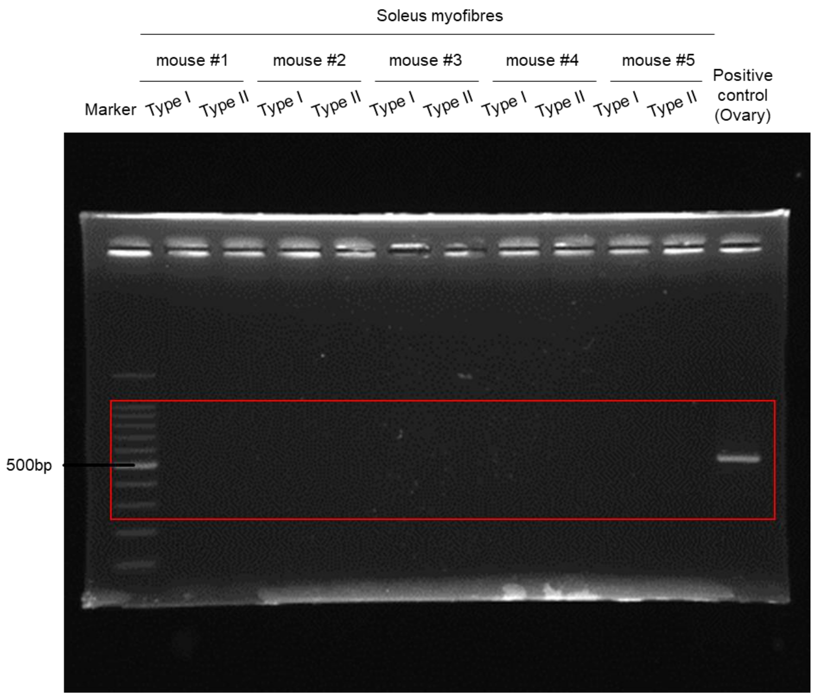

Rspo1

Supplementary Fig. 2

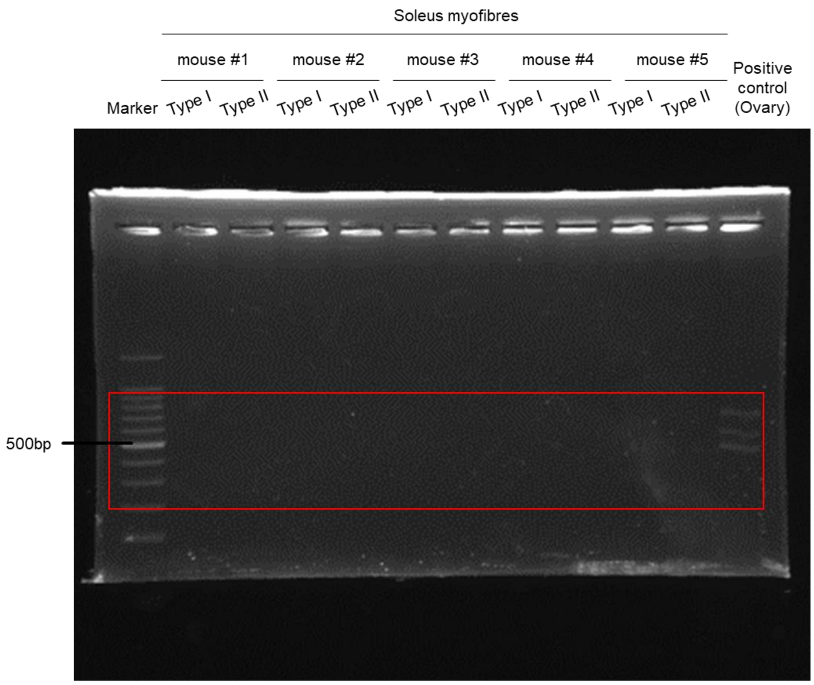

Rspo2

(*legend on the following pages*)

Supplementary Fig. 2

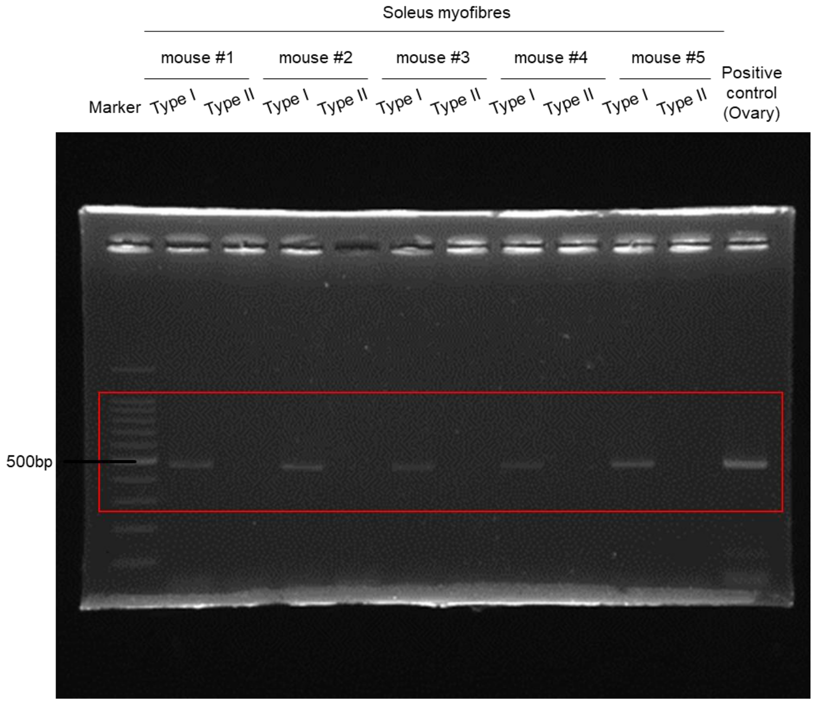

Rspo3

Supplementary Fig. 2

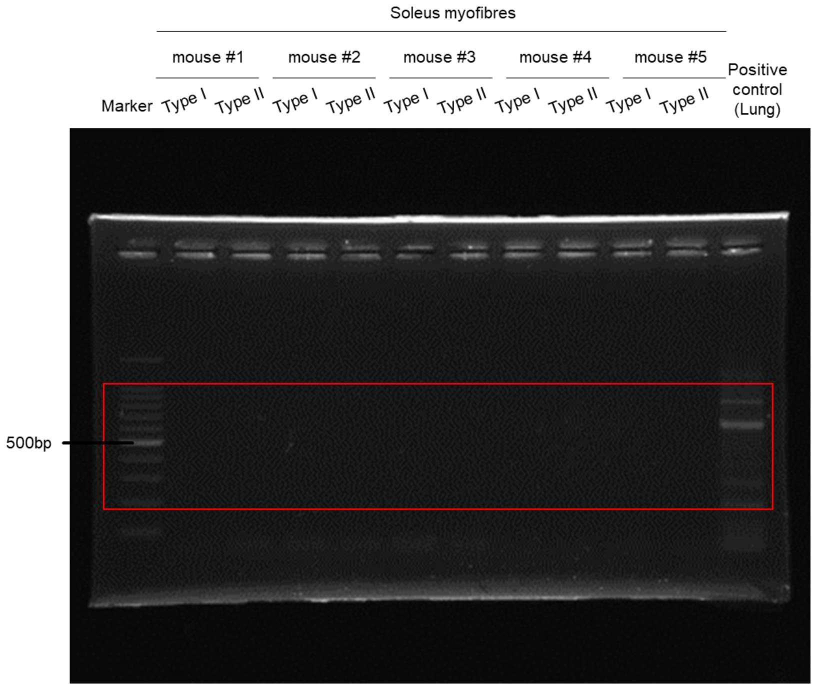

Rspo4

(*legend on next page*)

Supplementary Fig. 2

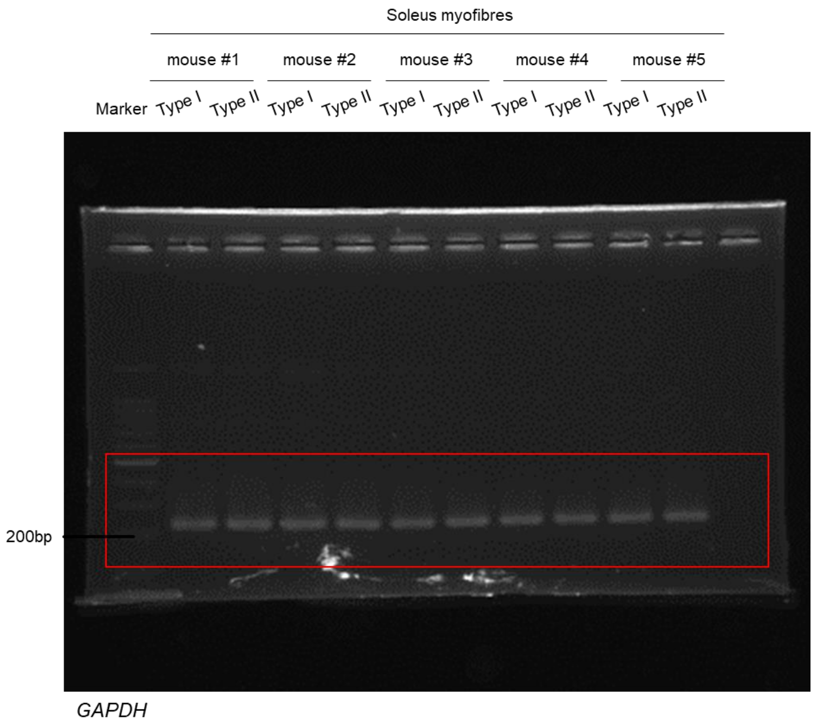

Supplementary Figure 14. Uncropped images of gel panels in Supplementary Figure 2.

The cropped images are outlined in red.

Supplementary Fig. 3A

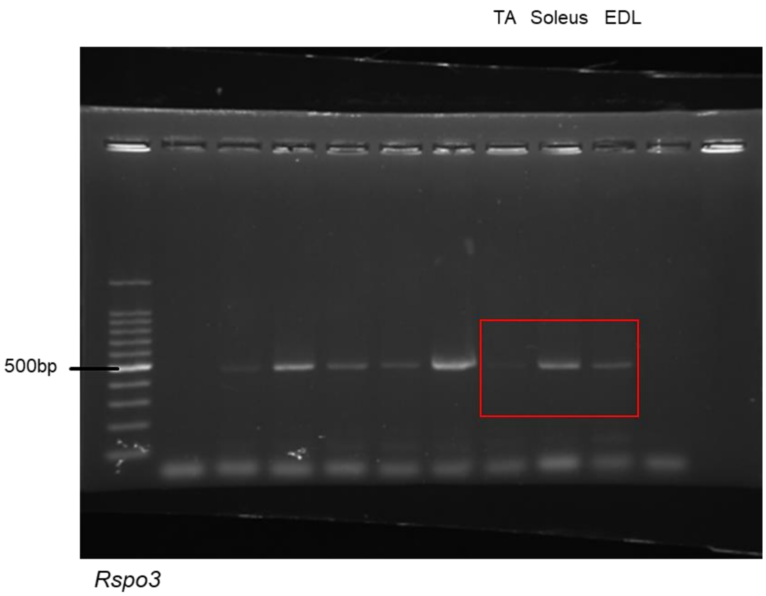

Supplementary Fig. 3A

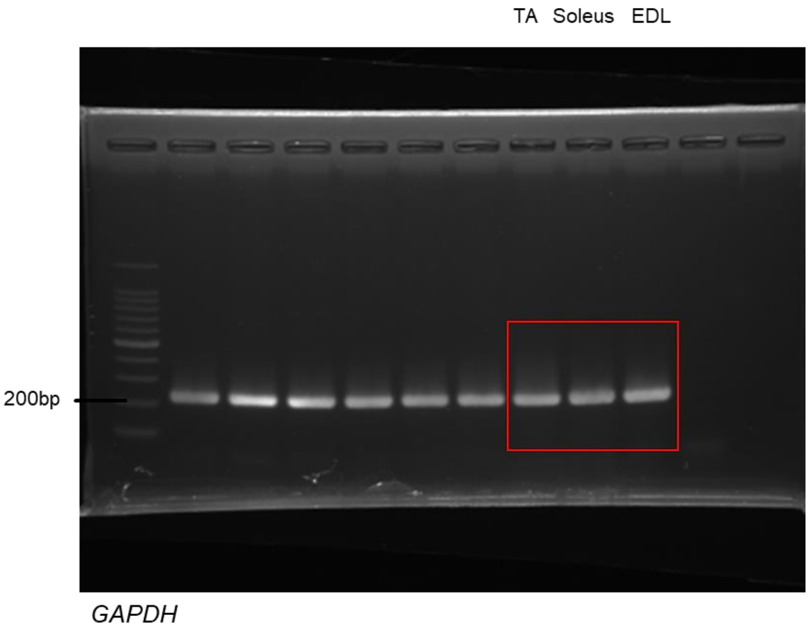

Supplementary Figure 15. Uncropped images of gel panels in Supplementary Figure 3.

The cropped images are outlined in red. TA; Tibialis anterior, EDL; extensor digitorum longus.

Supplementary Fig. 4B

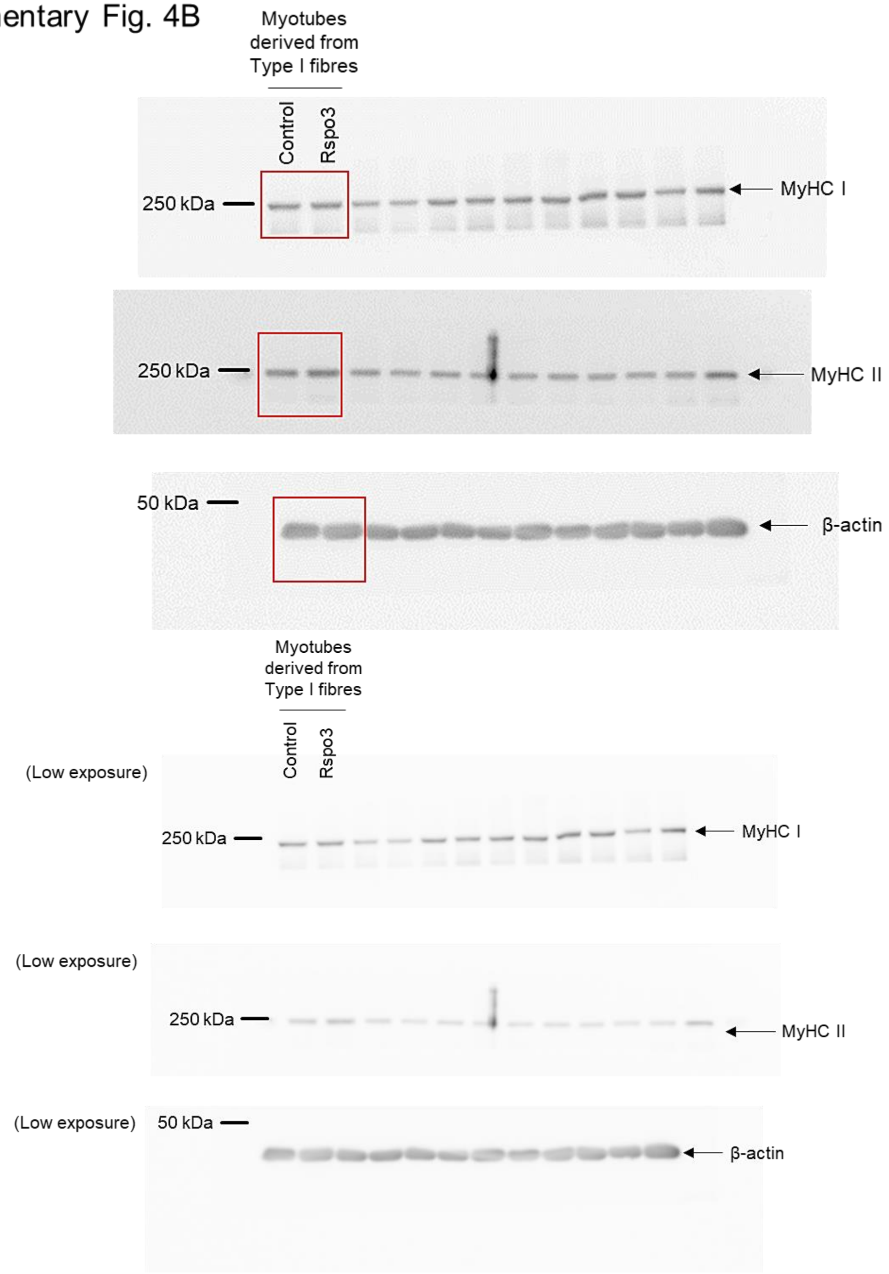

(*legend on next page*)

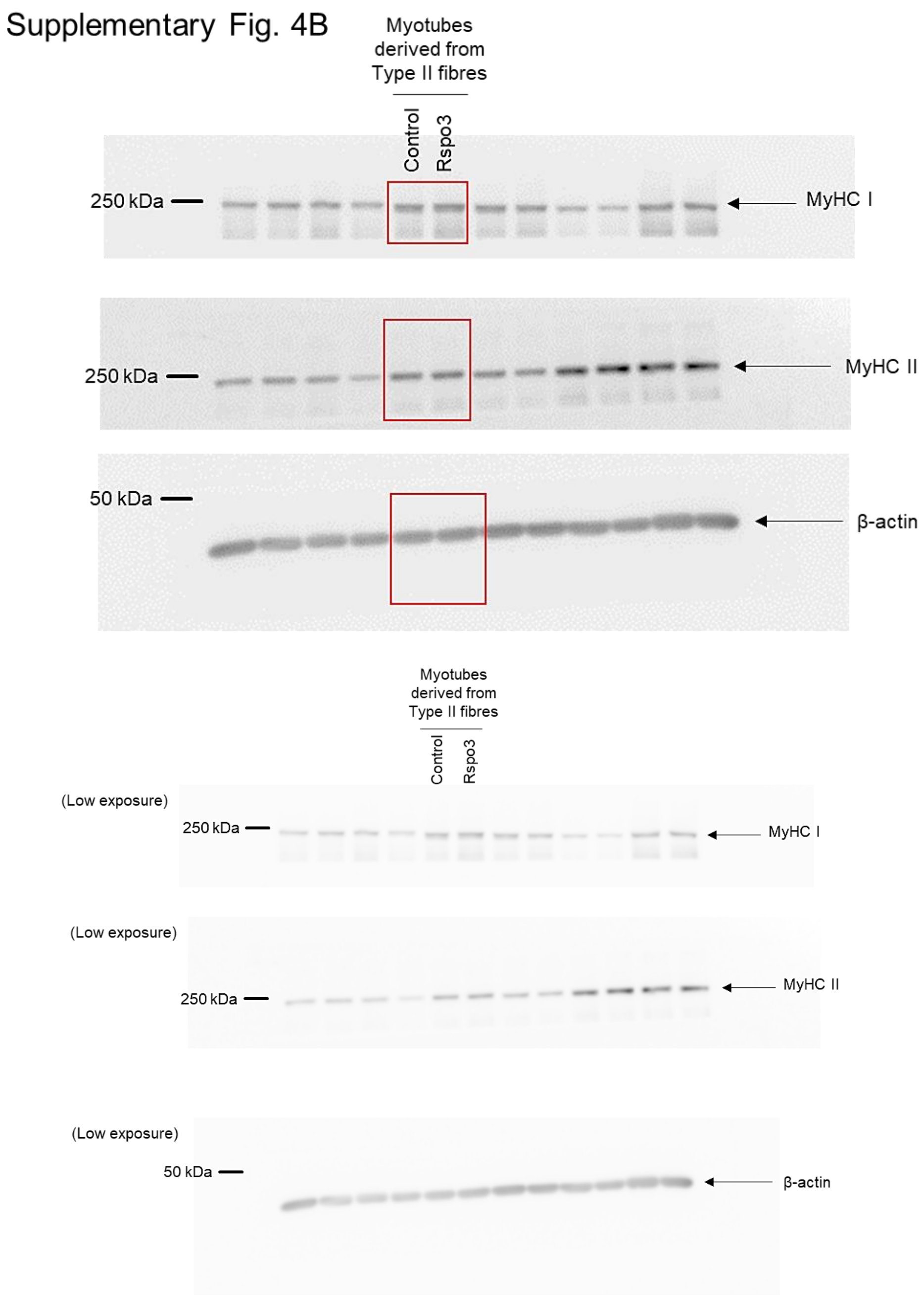

Supplementary Figure 16. Uncropped images of Western blot in Supplementary Figure 4.

The cropped images are outlined in red.

Supplementary Fig. 5

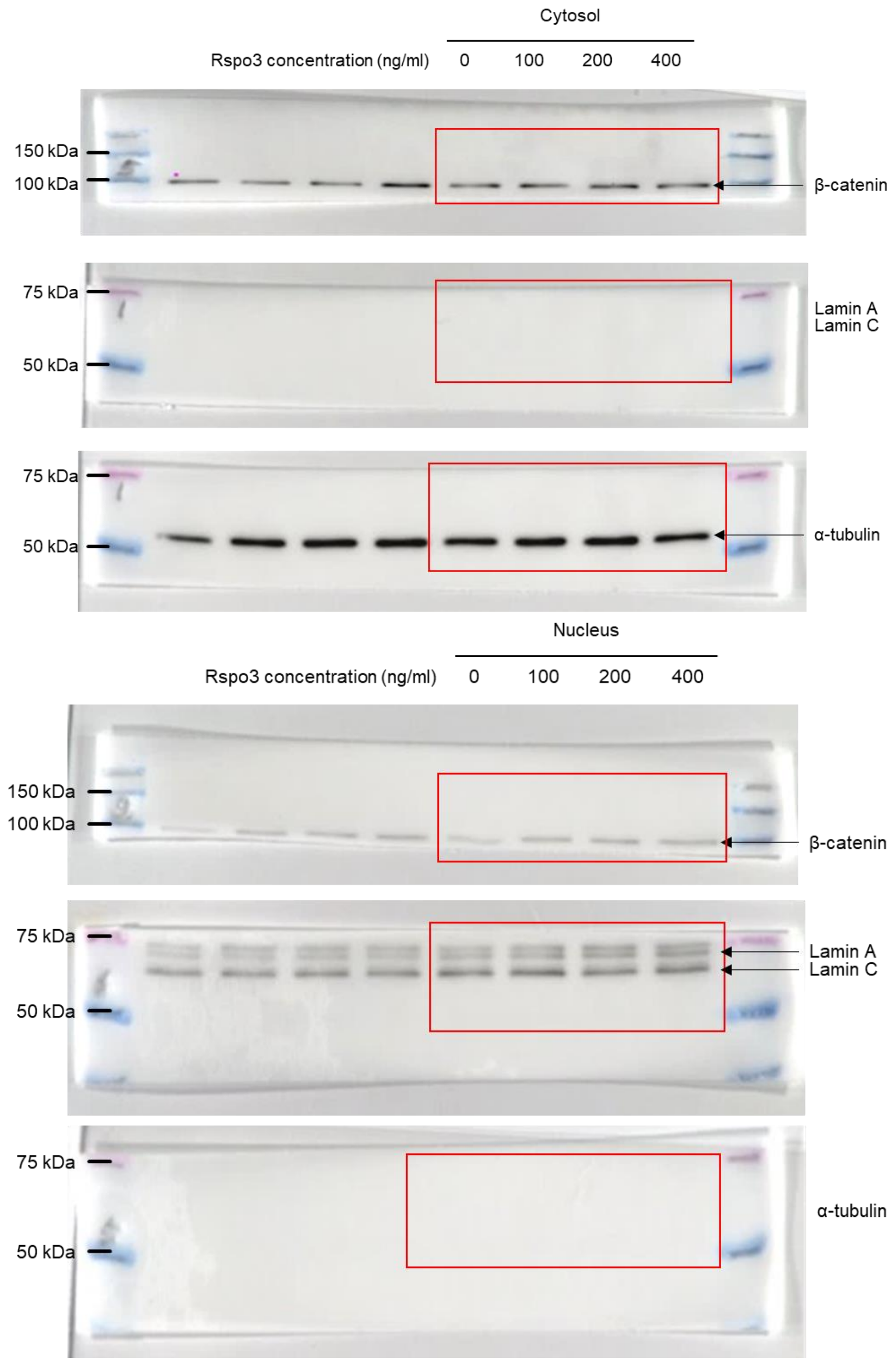

Supplementary Figure 17. Uncropped images of Western blot in Supplementary Figure 5.

The cropped images are outlined in red.

Supplementary Fig. 6B

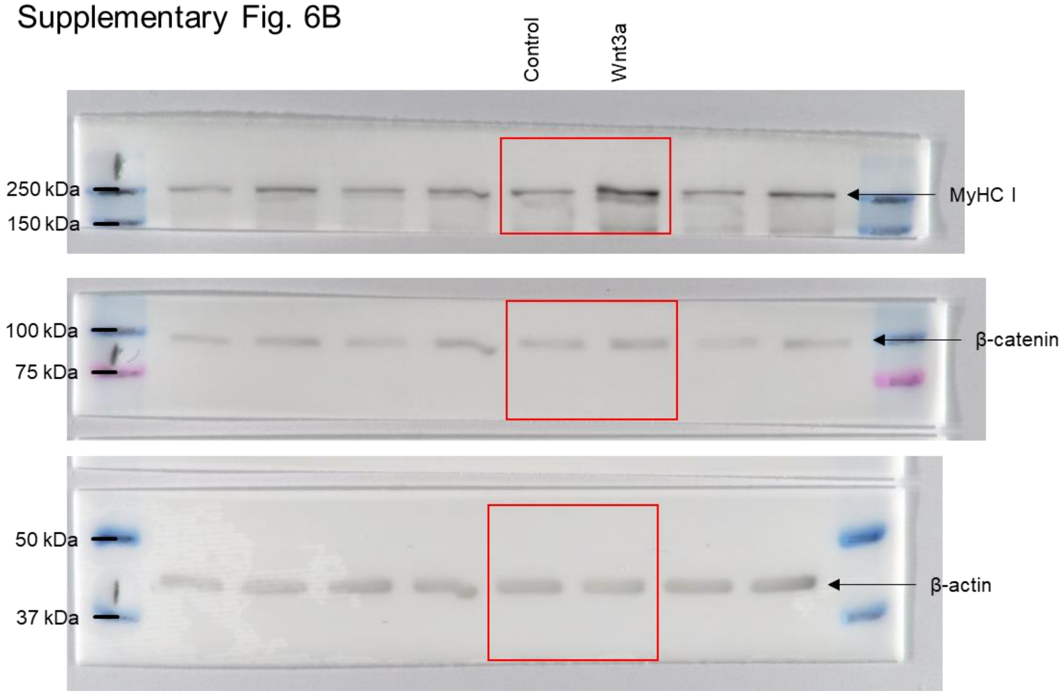

Supplementary Figure 18. Uncropped images of Western blot in Supplementary Figure 6.

The cropped images are outlined in red.

Supplementary Table 1: Sequences of the primers used for conventional PCR in this study.

Gene	Forward (5' – 3')	Reverse (5' – 3')
Rspo1	GCTGGCGTGAGAAGACTTCT	GCCTCACAGTGCTCGATCTT
Rspo2	CGCTCGGCTGAAGCTAGTTA	CTCCTGGACAGTGCCTCATG
Rspo3	GTACACTGTGAGGCCAGTGAA	ATGGCTAGAACACCTGTCCTG
Rspo4	TGGACATGCTCGCCCTGTA	CGCCGATCCTTCCTGTTCTT
GAPDH	AACTTTGGCATTGTGGAAGG	ACACATTGGGGGTAGGAACA

*All sequences are sequences for mice.

Supplementary Table 2: Sequences of the primers used for quantitative real-time PCR in this study.

Gene	Forward (5' – 3')	Reverse (5' – 3')
Rspo3	TGTCAGTATTGTACACTGTGAGGC	AGTTCTTGTCTCGCTGGTTGG
Tbp	AATGACTCCTATGACCCCTATCAC	AGGTCAAGTTTACAGCCAAGATTC

*All sequences are sequences for mice.

Supplementary Table 3: Experimental reagents and antibodies.

Reagent resource	Source	Identifier	
**Antibodies**			**Dilution**
Monoclonal Anti-Myosin (Skeletal, Slow) antibody produced in mouse	Sigma-Aldrich	Cat# M8421, RRID: AB_477248	WB (1:1000) IHC (1:600)
Monoclonal Anti-Myosin (Skeletal,	Sigma-Aldrich	Cat# M4276, RRID: AB_477190	WB (1:1000) IHC (1:600)

Fast) antibody produced in mouse			
β-Actin Antibody	Cell Signaling Technology	Cat# 4967, RRID: AB_330288	WB (1:1000)
Mouse Anti-alpha-Tubulin Monoclonal Antibody, Unconjugated, Clone DM1A	Cell Signaling Technology	Cat# 3873, RRID: AB_1904178	WB (1:1000)
Lamin A/C (4C11) Mouse mAb	Cell Signaling Technology	Cat# 4777, RRID: N/A	WB (1:2000)
β-Catenin Antibody	Cell Signaling Technology	Cat# 9562, RRID: AB_331149	WB (1:1000)
Phospho-SAPK/JNK (Thr183/Tyr185) (81E11) Rabbit mAb antibody	Cell Signaling Technology	Cat# 4668, RRID: AB_823588	WB (1:1000)
SAPK/JNK Antibody	Cell Signaling Technology	Cat# 9252, RRID: AB_2250373	WB (1:1000)
NFATc1 (7A6) antibody	Santa Cruz Biotechnology	Cat# sc-7294, RRID: AB_2152503	WB (1:500)
Mouse Anti-Human Myosin Heavy Chain Monoclonal Antibody,	R&D Systems	Cat# MAB4470, RRID: AB_1293549	ICC (1:400)

Unconjugated, Clone MF20			
Desmin antibody [Y266]	Abcam	Cat# ab32362, RRID: AB_731901	ICC (1:100)
Donkey Anti-Rabbit IgG, Whole Ab ECL Antibody, HRP Conjugated	GE Healthcare	Cat# NA934, RRID: AB_772206	WB (1:1000)
Sheep Anti-Mouse IgG - Horseradish Peroxidase antibody	GE Healthcare	Cat# NA931, RRID: AB_772210	WB (1:1000)
Goat anti-Mouse IgG (H+L) Cross-Adsorbed Secondary Antibody, Alexa Fluor 488	Thermo Fisher Scientific	Cat# A-11001, RRID: AB_2534069	IHC (1:400)
Goat anti-Rabbit IgG (H+L) Cross-Adsorbed Secondary Antibody, Alexa Fluor 594	Thermo Fisher Scientific	Cat# A-11012, RRID: AB_141359	IHC (1:400)
**Chemicals and Recombinant proteins**			
Recombinant Mouse R-Spondin 3 Protein	R&D Systems	Cat# 4120-RS-025	
Recombinant Mouse	R&D Systems	Cat# 1324-WN-002	

Wnt-3a Protein		
XAV-939	Chemscene LLC	Cat# CS-0494
